# Improving reproducibility and performance of radiomics in low‐dose CT using cycle GANs

**DOI:** 10.1002/acm2.13739

**Published:** 2022-07-30

**Authors:** Junhua Chen, Leonard Wee, Andre Dekker, Inigo Bermejo

**Affiliations:** ^1^ Department of Radiation Oncology (MAASTRO), GROW School for Oncology and Developmental Biology Maastricht University Medical Centre+ Maastricht ET Netherlands

**Keywords:** computed tomography, cycle GAN, denoising, radiomics, reproducibility

## Abstract

**Background:**

As a means to extract biomarkers from medical imaging, radiomics has attracted increased attention from researchers. However, reproducibility and performance of radiomics in low‐dose CT scans are still poor, mostly due to noise. Deep learning generative models can be used to denoise these images and in turn improve radiomics’ reproducibility and performance. However, most generative models are trained on paired data, which can be difficult or impossible to collect.

**Purpose:**

In this article, we investigate the possibility of denoising low‐dose CTs using cycle generative adversarial networks (GANs) to improve radiomics reproducibility and performance based on unpaired datasets.

**Methods and materials:**

Two cycle GANs were trained: (1) from paired data, by simulating low‐dose CTs (i.e., introducing noise) from high‐dose CTs and (2) from unpaired real low dose CTs. To accelerate convergence, during GAN training, a slice‐paired training strategy was introduced. The trained GANs were applied to three scenarios: (1) improving radiomics reproducibility in simulated low‐dose CT images and (2) same‐day repeat low dose CTs (RIDER dataset), and (3) improving radiomics performance in survival prediction. Cycle GAN results were compared with a conditional GAN (CGAN) and an encoder–decoder network (EDN) trained on simulated paired data.

**Results:**

The cycle GAN trained on simulated data improved concordance correlation coefficients (CCC) of radiomic features from 0.87 (95%CI, [0.833,0.901]) to 0.93 (95%CI, [0.916,0.949]) on simulated noise CT and from 0.89 (95%CI, [0.881,0.914]) to 0.92 (95%CI, [0.908,0.937]) on the RIDER dataset, as well improving the area under the receiver operating characteristic curve (AUC) of survival prediction from 0.52 (95%CI, [0.511,0.538]) to 0.59 (95%CI, [0.578,0.602]). The cycle GAN trained on real data increased the CCCs of features in RIDER to 0.95 (95%CI, [0.933,0.961]) and the AUC of survival prediction to 0.58 (95%CI, [0.576,0.596]).

**Conclusion:**

The results show that cycle GANs trained on both simulated and real data can improve radiomics’ reproducibility and performance in low‐dose CT and achieve similar results compared to CGANs and EDNs.

## 1 INTRODUCTION

Biomarkers from medical imaging can provide a macroscopic view of the tissue of interest and can be an effective tool to accurately diagnose disease in precision medicine.^1^ Radiomics features^2^ have shown value as potential imaging biomarkers in various tumor and neurodegenerative diseases, such as lung cancer,^3^ head and neck cancer,^4^ rectal cancer,^5^ breast cancer,^6^ Alzheimer disease,^7^ and autism spectrum disorder.^8^

However, in computed tomography (CT), the repeatability and reproducibility of radiomics have been challenged in multiple published studies.^9^, ^10^, ^11^, ^12^ The reproducibility of radiomics can be impacted by various CT parameters such as radiation dose, slice thicknesses, and reconstruction algorithm settings. More specifically, it has been reported that only 11.3% (12 of 106) of radiomics features are robust to these technical parameters.^12^ In fact, slice thickness ranks first on impact on radiomics’ reproducibility, while signal‐to‐noise ratio ranks second. Intensity and texture radiomic features are especially sensitive to radiation dose and the associated signal‐to‐noise ratio.^12^ Therefore, it is likely that radiomic features extracted from low‐dose CT are less accurate than features from high‐dose CT. In other words, radiomics applied to low‐dose CT will likely have low reliability, and thus the established radiomics signature or models are likely to have worse performance compared to high‐dose CT.^13^

In this study, we aim to use denoising^14^ to improve the reliability of radiomics in low dose CT. A variety of image denoising methods have been proposed in the past several decades, and these methods can be divided into two classes—model‐based denoisers^15^, ^16^ and data‐driven denoisers.^17^, ^18^ Multiple published studies^18^, ^19^ have demonstrated that data‐driven denoisers outperform model‐based denoisers and achieve state‐of‐art denoising quality when suitable training datasets are available.

Most data‐driven denoisers are based on deep convolutional neural networks (DCNNs)^20^ in which this denoising task is posed as an image‐to‐image translation problem. The popular architectures for medical image denoising are full convolutional network (FCN),^21^ encoder–decoder network (EDN),^22^ and generative adversarial networks (GAN)^23^ which were described in detail in recent reviews.^14^, ^24^ An important characteristic of most data‐driven denoisers is that datasets consisting of paired low‐high dose CTs from the same subjects are needed to train the deep neural networks. However, collecting paired low‐high dose CT is time‐consuming, expensive, and impossible in many cases, for example, in patient studies.

Therefore, it is the aim of this study to establish a CT denoiser based on unpaired datasets to improve radiomics performance. The related literature is divided into two topics—low‐dose CT denoising and radiomics normalization. In this section, we review these two topics briefly.

(a) Low‐dose CT denoising

As mentioned above, most data‐driven denoisers are based on one of three backbones—FCN, EDN, and GAN—and all of them are used in low‐dose CT denoising tasks. More specifically, Yang et al.^25^ used a 3D residual network as the denoising network architecture with a loss function based on differences between the ground truth residual image and reconstructed residual image. Moreover, pool layers were removed from the network to generate denoised residual images because there is no size or resolution change between input and output. The results show that the network can reduce noise effectively while preserving tissue details. Chen et al.^26^ adapted an EDN as the backbone of their denoiser, and two residual shortcuts were added into the network to keep details of the image from encoder to decoder. Models were trained by using simulated data, and the trained denoiser achieved a competitive performance in both simulation and clinical cases. Yang et al.^27^ took conditional GAN (CGAN)^28^ as the backbone where they replaced Jensen–Shannon divergence^29^ with Wasserstein distance^30^ to measure the differences in the data distribution. Moreover, Yang et al. replaced the mean squared error (MSE) loss function with Perceptual Loss^31^ to keep more texture information from low‐dose CT to high‐dose CT. They proposed a method to not only reduce the image noise level but also tried to keep the critical information at the same time.

One of the biggest shortcomings of these aforementioned denoisers is that paired low‐high dose datasets are needed in denoiser training. However, collecting this kind of datasets is time consuming and expensive. As an alternative, a few simulation paired low–high dose CT datasets are publicly available, such as the dataset from 2016 NIH‐AAPM‐Mayo Clinic Low Dose CT Grand Challenge (LDGC).^32^ The low‐dose CT images in this dataset are simulated data with a simulated low radiation dose of 50 mAs. The characteristics of LDGC dataset decrease the value for network training, as the generalization of models trained from the LDGC to real low‐dose CT is questionable because the exposure in real low‐dose CT datasets will be much lower than the simulated data in LDGC. For example, radiation dose in The Reference Image Database to Evaluate Therapy Response (RIDER)^33^ ranged from 7 mAs to 13 mAs.

Therefore, we believe that implementing a denoiser based on unpaired datasets could help to relieve the problem of data collection and make unsupervised CT denoising for quantitative medical image analysis possible. There are a few studies that used this strategy; Kang et al.^34^ used cycle GAN as the backbone for multiphase coronary CT angiography correction where they took routine‐dose CT from multiphase coronary CT angiography as the target domain data and low‐dose CT as the original domain data to build a training dataset. The results show that visual grading and quality evaluation of low‐dose CT are improved; however, they did not investigate the effect of cycle GANs into deeper quantitative metrics such as radiomics.

However, to the best of our knowledge, there are no studies that apply unsupervised CT denoising to improve radiomics reliability and reproducibility in low‐dose CT.

(b) Radiomics normalization

Berenguer et al.^10^ have shown that over half of radiomics features are nonreproducible when images scanned from different scanners even when using the same CT parameters. The results of radiomics signatures or models, which based on nonreproducible features are thus unreliable. Li et al.^35^ used cycle GAN to normalize CT images from multiple centers and multiple scanners, and then they extracted features from normalized images and established radiomics signatures. They found the average improvement of a classifier based on normalized radiomics features in the area under the receiver operating characteristic curve (AUC) to be 11%. Yang et al.^36^ integrated adaptive instance normalization (AdaIN) into cycle GAN for continuous CT kernel conversion, introduced AdaIN kept more content information from original domain to target domain. The proposed method is promising for radiomics normalization in different CT kernels. The major difference between previous studies, and our study is that this paper focused on using cycle GAN to improve radiomics reproducible and performance in low dose CTs.

In previous work,^13^ we used EDN and CGAN^37^ as testing backbones to denoise low‐dose CT. Our training datasets consisted of paired simulated low‐dose CT and high‐dose CTs. Radiomics features reproducibility from noisy images and denoised images were measured using concordance correlation coefficients (CCC).^38^ The results showed that EDN and CGAN can improve CCC of noisy images significantly. Moreover, when we applied our trained denoisers to real low‐dose CT images (RIDER dataset); the results showed that this denoiser can improve radiomics reproducibility in realistic low‐dose CTs.

In another study,^39^ we applied the trained denoisers to improve radiomics performance in realistic applications. The results showed that generative models based denoisers can improve the AUC of a lung cancer survival prediction from 0.52 (95%CI, [0.511,0.538]) to 0.58 (95%CI, [0.564,0.596]) and a multiple instance learning‐based lung cancer diagnostic^40^ from 0.84 (95%CI, [0.828,0.856]) to 0.88 (95%CI, [0.866,0.892]).

The major shortcoming of our previous studies is that denoising models were exclusively dependent on paired simulated data, which may cause the trained denoiser to not generalize well to real data. In this paper, we took cycle GAN as basic denoising model to train a denoiser using unpaired low–high dose CT. These low‐ and high‐dose CT images were collected from different centers and scanners. We evaluated this new denoiser for its ability to improve radiomics reproducibility and performance in realistic applications.

In comparison with previous studies, the major contribution of this study is that we assess the potential of denoising low‐dose CTs using cycle GANs based on unpaired data to improve radiomics reproducibility and performance. The results show that cycle GANs can improve radiomics’ reproducibility and performance in low‐dose CT and achieve similar results compared to CGANs and encoder–decoder networks. Source code, Radiomics features, data for statistical analysis, and Supporting Information of this article are online at https://gitlab.com/UM‐CDS/low‐dose‐ct‐denoising/‐/tree/Cycle_GAN_Improve_Radiomics.

## 2 MATERIALS AND METHODS

In this section, we describe the architecture and technical details of our cycle GAN. Then, we introduce our training strategy to improve the speed of convergence. Next, we describe the design of the experiments and datasets used for training and testing. Finally, we describe the extraction of the radiomics features and the evaluation metrics used.

### 2.1 Cycle GANs

**FIGURE 1:**
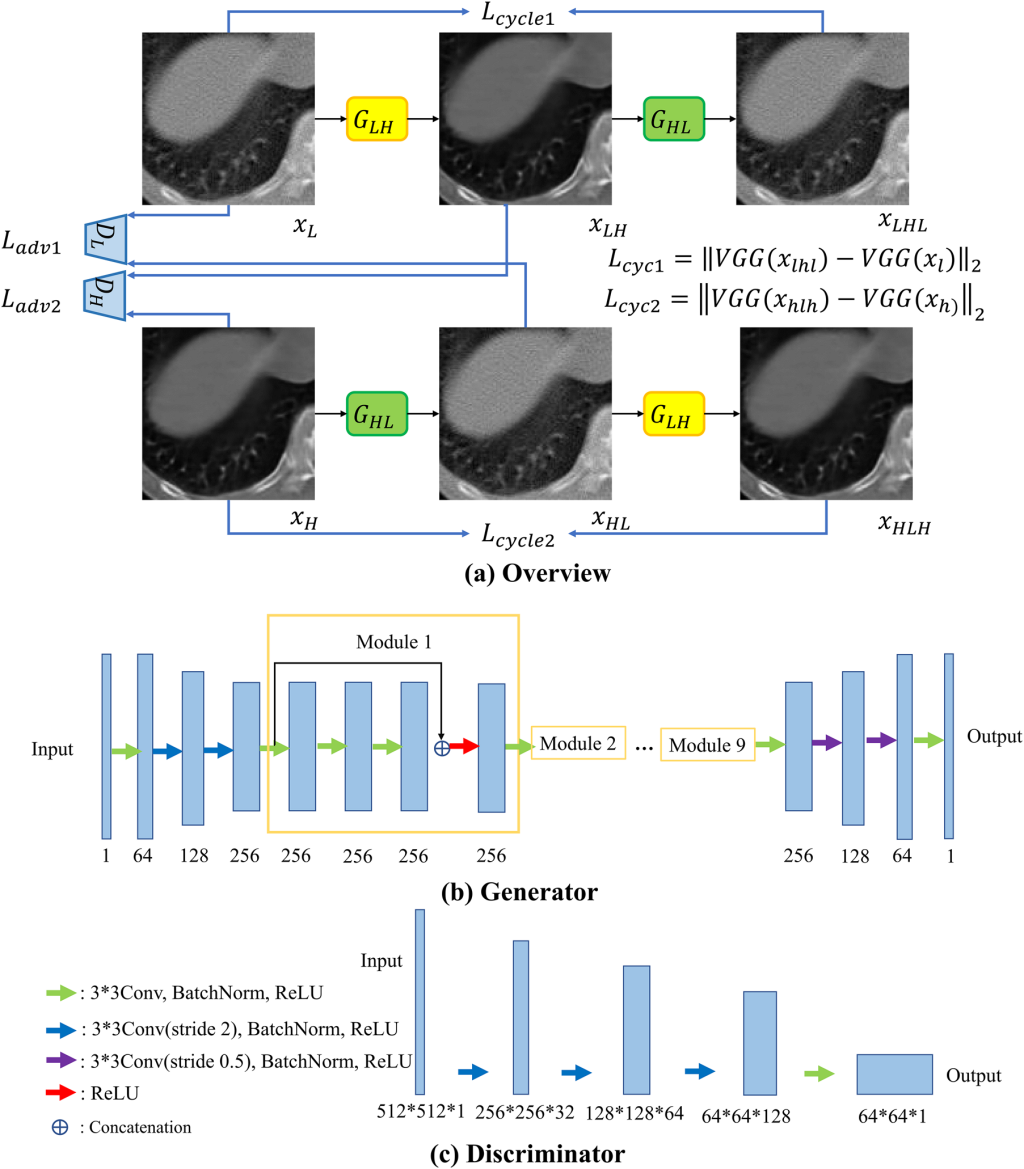
Overview of network, architecture of generator, and discriminator

We use cycle‐consistent GANs, proposed by Zhu et al.^41^ As shown in Figure 1a, the cycle GAN consist of two generators and two discriminators. The generator $G_{LH}$ maps from low‐dose CT domain (*L*) to full dose CT domain (*H*), while $G_{HL}$maps fromH to *L*. The loss function of the cycle GAN consists of two parts—adversarial loss and cycle consistency loss, represented with $L_{adv}$ and $L_{cyc}$ respectively (and each of them can be broken down into $L_{adv1},L_{adv2}$ and $L_{cyc1}L_{cyc2}$, one for each generator). The adversarial loss for mapping from low‐dose to full dose CT is defined as follows:

(1) $$\begin{array}{ccc} & & L_{adv1}\;\left(G_{LH},D_{H},L,H\right)=E_{x_{h}∼pdata\left(x_{h}\right)}\;\left[logD_{H}\left(x_{h}\right)\right]\\ & & +\;E_{x_{l}∼pdata\left(x_{l}\right)}\left[log(1-D_{H}\left(G_{LH}\left(x_{l}\right)\right)\right] \end{array}$$

where $G_{LH}$ is trained to transform low‐dose CT image $x_{l}$ to into high‐dose CT image $x_{h}$ (denoising), while $D_{H}$ is trained to discriminate between denoised CT images $G_{LH}\left(x_{l}\right)$ ($x_{LH}$ in Figure 1a) and real high‐dose CT image $x_{H}$. During the training, *G* aims to minimize this loss function against an adversary *D* that tries to maximize it; therefore, Equation (1) can be rewritten as follows:

(2) $$\begin{array}{ccc} & & min_{G}max_{D}L_{adv1}\;\left(G_{LH},D_{H},L,H\right)\\ & & =E_{x_{h}∼pdata\left(x_{h}\right)}\;\left[logD_{H}\left(x_{h}\right)\right]\\ & & +\;E_{x_{l}∼pdata\left(x_{l}\right)}\left[log(1-D_{H}\left(G_{LH}\left(x_{l}\right)\right)\right] \end{array}$$

The definition of adversarial loss for mapping from high‐dose CT to low‐dose CT is defined in similar way, and we denote it as $min_{G}max_{D}L_{adv2}\left(G_{HL},D_{L},H,L\right)$. Moreover, we denote the adversarial loss for the whole network as $L_{adv}\;\left(G,D\right)=L_{adv1}\;+L_{adv2}$.

Regarding the cycle consistency loss of our cycle GAN, we replace the mean squared error (MSE) loss function used in the original cycle GAN with a perceptual loss‐based loss function. The definition of cycle consistency loss is as follows:

(3) $$\begin{array}{ccc} & & L_{cyc1}=\\ & & E\left(x_{l},x_{lhl}\right)\left[\frac{1}{wed}∥VGG\left(G_{HL}\left(x_{lh}\right)\right)-VGG\left(x_{l}\right){∥}^{2}\right] \end{array}$$

where $x_{l}$ represents lowdose CT image and $x_{lhl}$ represents reconstructed low‐dose CT image from fake synthetic high‐dose CT image, *w, e*, and *d* represent width, height, and depth of the feature map, and VGG(.) represents feature maps from a pre‐trained VGG‐16 at a specific convolutional layer. VGG‐16 is pre‐trained on ImageNet,^42^ a dataset of over 14 million images belonging to 1000 classes. In order to feed CT images into a model pretrained on color images, they need to be triplicated into RGB channels before cycle consistency loss calculation. In our implementation, we select feature maps from conv2_1 to calculate perceptual loss. $L_{cyc2}$ can be defined in similar way with $G_{LH}$. We denote $L_{cyc1}+L_{cyc2}$ as $L_{cyc}\left(G\right)$.

Combining Equations (2) and (3), the overall loss function is expressed as:

(4) $$min_{G}max_{D}L_{adv}\left(G,D\right)+\lambda L_{cyc}\left(G\right)$$

where λ is a parameter to control the trade‐off between the adversarial and perceptual loss.

More details about the architecture of generators and discriminators can be found in Figure 1b,c, respectively.

### 2.2 Slice‐paired training strategy

Randomly chosen samples from two domains are fed to the networks in training a cycle GAN. However, as mentioned in the original cycle GAN article,^41^ the training will be more successful and stable when focusing on pairs of visually similar images.

In the case of CT scans, assuming all scans belong to the same organ (the lung in our case), we can expect that images belonging to the same slice number will be more similar to each other than images from different slices. Hence, the first slice of a low‐dose CT scan will have higher similarity with the first slice of a high‐dose CT scan.

Therefore, CT‐based cycle GAN training should be fed with pairs of the same (randomly chosen) slice rather than images of different slices. This could be seen as weakly supervised learning. We call this strategy slice‐paired training strategy hereafter, a similar training strategy can be found in literature.^43^

### 2.3 Data acquisition

In order to compare results of cycle GANs with our previous work (CGAN and EDN),^13^, ^39^ we trained networks on the same data as used in^13^, ^39^ and applied the trained models to the same applications on the same datasets. In total, we used six datasets in this study.

We used a phantom dataset to test whether our GANs generated artifacts when denoising.^44^ This phantom dataset is a collection of phantoms CTs by scanning a Gammex 467 CT phantom (Middleton, WI, USA) using a Philips Brilliance Big Bore CT with different doses (50 mAs, 400 mAs). CT images scanned at 50 mAs are referred to as low‐dose CT and 400 mAs referenced as high‐dose CT. We used 52 paired images from two scans for testing.

The second is based on the NSCLC‐Radiomics dataset (hereafter called LUNG 1).^45^ We selected only the high‐dose CT scans, those scanned at 400 mAs or more (*n* = 157, indices in Table S1) and added noise to the sinograms to simulate low‐dose CTs with two different levels of noise: low‐noise CT and high‐noise CT. The specific methods used to add noise are described in^13^ section 2.3 and in the Method S1. We used a subset of these high‐noise CTs and their corresponding high dose CTs (40 subjects, 4260 images) to train a cycle GAN, and we used the remaining images to assess the reproducibility of radiomics features in the original high‐dose CT versus those in the denoised images.

The third and fourth datasets were used to train the cycle GAN with real low dose CT scans. We used low dose CT scans from the Lung Image Database Consortium dataset (LIDC‐IDRI),^46^ and high‐dose CT scans from The Cancer Genome Atlas Lung Adenocarcinoma (TCGA‐LUAD) dataset.^47^ We used two inclusion criteria for CTs in both datasets to increase the visual similarity across the two domains: the use of GE scanner; table height ranging from 150 mm to 160 mm. As low‐dose CTs, we included those with a radiation exposure lower than 10 mAs and as high dose CTs those with and exposure higher than 100 mAs (list of indices of selected samples is in Tables S2 and 3 respectively). Examples of selected samples from LIDC‐IDRI and TCGA‐LUAD are shown in Figure S1.

The final two datasets, used for the two radiomics‐based applications, are RIDER^33^ and NSCLC Radiogenomics.^48^ RIDER is a collection of same day repeat CT scans collected to assess the variability of tumor measurements, which makes it particularly useful to assess the reproducibility of radiomics across pairs of similar CT scans. We use the trained cycle GAN to denoise the images in RIDER to assess the impact of denoising on the reproducibility of radiomic features. NSCLC Radiogenomics is a radiogenomic dataset from a cohort of 211 patients with non‐small cell lung cancer,^48^ from which we selected the low‐dose CT images, their respective segmentation masks and clinical data for survival prediction (*n* = 106), the indices of the included samples are included in Table S4. The average radiation exposure of samples selected from NSCLC Radiogenomics is 38.65 ± 81.97 mAs (±standard error of the mean, SEM) (the distribution of radiation exposure for selected samples can be found in Figure S2).

A summary of scanning parameters of included datasets is shown in Table 1.

**TABLE 1:** Scanning parameters of included datasets

Parameters				
Datasets	Scanner	Radiation dose(mAs)	Slice thickness(mm)	Spatial resolution(mm)
Phantom Dataset	Philips (4^a^)	50 (2), 400 (2)	3 (4)	[0.77,0.77]
LUNG 1	Siemens (157)	400 (157)	3 (157)	[0.98,0.98]
TCGA‐LUAD	Siemens (14)	110 (3), 120 (8), 140 (2), 210 (1)	1 (2),5 (8), 8 (4)	[0.59,0.59]‐[0.74,0.74]
LIDC‐IDRI	GE (12)	< 1 (12)	1.25 (7),2.5 (5)	[0.53,0.53]‐[0.70,0.70]
RIDER	N/A^b^ (56)	4 (4), 5( 4),6 (6), 7 (13), 8 (13), 9 (9), 10 (7)	1.25 (56)	[0.51,0.51]‐[0.82,0.82]
NSCLC Radiogenomics	N/A (5), Philips (1), GE (90), Siemens (10)	38.65 ± 81.97	0.625 (7), 1 (11), 2 (1), 1.25 (75), 2.5 (9), 3 (2)	[0.59,0.59]‐[0.98,0.98]

*Note*: ± standard error of the mean.

^a^Number of included scans.

^b^Manufacturer not mentioned in DICOM metadata

### 2.4 Experiments

We trained three cycle GANs to denoise low CT scans: on a paired dataset with low‐dose CT scans simulated from high‐dose CT scans with and without the Slice‐paired training strategy (referred to as ablation study hereafter) and on unpaired real low and high dose CT scans. Regarding CT normalization, the CT HU was set to ‐1000 when it was lower than −1000 to 1000 when it was higher than 1000, and then normalized to intensity [0,1] for network training and image denoising.

Then, we assessed the performance of the denoising using root mean square error (RMSE) and perceptual loss as evaluation metrics. The definition of perceptual loss can be found in Equation (3) and definition of RMSE is as follows:

(5) $$RMSE\;=\sqrt{\frac{1}{M}\sum_{i=1}^{M}{\left(y_{i}-{\hat{y}}_{i}\right)}^{2}}\;$$

where $y_{i}$ and ${\hat{y}}_{i}$ represent the image value in position *i* for the original high‐dose CT and denoised CT, respectively. Image values were normalized to 0–1 before calculating RMSE. M represents the number of pixels in one image, 512 × 512 in our case.

We also assessed the impact of denoising on reproducibility of radiomic features by calculating the concordance correlation coefficients (CCC)—a metric that measures the degree of agreement between two variables (e.g., to evaluate reproducibility or for inter‐rater reliability) as defined in.^38^ Several arguments support our choice of CCC as the reproducibility metric: according to a recent systematic review,^49^ CCC is the most common metric used to measure the reproducibility of radiomics. Moreover, the seminal article that introduced the CCC^38^ has shown the clear advantages of using this metric in testing reproducibility in comparison with other methods. On the simulated paired data, we calculated the CCCs of the radiomic features extracted in the original high dose CT and the denoised CT. In RIDER, we calculated the CCC of the same day denoised CT scans.

In the ablation study, we assessed the impact of using the position‐based training strategy comparing the performance in terms of RMSE, perceptual loss, and CCC on synthetic data.

Next, we applied the trained cycle GAN to two applications—radiomics reproducibility in same‐day repeat CT scans and pre‐treatment survival prediction—without retraining. Pretreatment survival prediction of cancer patients is a typical application of radiomics since it appeared in the seminal article by Aerts et al.^2^ We predicted pretreatment survival in two different ways: as a binary outcome on 4‐year survival and as time‐to‐event continuous outcome. For the first, we used least squares support vector machines (SVMs) with radial basis function (RBF) Kernel as our classifier. For hyperparameter search and internal validation, we used 40‐repeat nested 5‐fold cross‐validation.^50^ More details on the survival prediction modeling can be found in.^39^ The metric used for measuring the performance this model is the area under the receiver operating characteristic curve (AUC).^51^ For the time‐to‐event survival analysis, we fitted a Cox proportional hazards models, using the radiomics features (103 features) as predictors. To ensure convergence during parameter fitting, we used penalized Cox regression with a penalty coefficient of 0.01. The discriminative performance of this model was measured using the concordance index (C‐index).

All experiments were implemented in Python 3.6 and TensorFlow 1.13.1. The training was run on one Nvidia Tesla V100 GPU 30.5GB of memory and 4 CPUs. We set λ in Equation (4) to 10 and the batch size to 1. The discriminator and the denoiser both used the Adam optimizer^52^ and shared the same learning rate. The initial learning rate was set to 0.0002 with a decay factor of 0.8 every 10 epochs. Training runs were stopped at 100 epochs and radiomics features were extracted every 25 epochs (i.e., at 25, 50, 75, and 100 epochs). No early stopping was adopted for terminating the model training. Table 2 offers a concise summary of our experiments.

**TABLE 2:** Summary of experiment and corresponding datasets

Experiment	Training strategy	Training dataset	Testing dataset
Simulated data‐based training	With slice‐pairing	Part of paired high‐noise and full dose Lung 1 dataset (*n* = 40, 4260 Frames)	The rest of high‐noise CTs (*n* = 117, 13,423 Frames), low‐noise CTs (*n* = 157, 17,683 Frames), phantom dataset CTs (*n* = 2, 104 Frames)
Ablation study	Without slice‐pairing	Part of paired high‐noise and full dose Lung 1 dataset (*n* = 40, 4260 Frames)	The rest of high‐noise CTs (n = 117, 13,423 Frames), low‐noise CTs (*n* = 157, 17,683 Frames)
Applications with simulated data training‐based networks	With slice‐pairing	Training finished at first part of experiment without re‐training	RIDER (*n* = 31, 14,875 Frames), NSCLC Radiogenomics (*n* = 106, 28,404 Frames)
Applications with real data training‐based networks	With slice‐pairing	Low dose CTs from LIDC‐IDRI (*n* = 12, 3144 Frames), Full dose CTs from TCGA‐LUAD (*n* = 14, 3307 Frames)	RIDER (*n* = 31, 14875 Frames), NSCLC Radiogenomics (*n* = 106, 28,404 Frames)

### 2.5 Radiomics extraction

The masks of the regions of interest (ROIs) are stored in DICOM format in 3D in the Lung 1, RIDER, and NSCLC Radiogenomics datasets. The modality of these files is “SEG.” DICOM CT images were converted to 3D images using the SimpleITK (v1.2.4) software. We resampled the images to 2‐mm isotropic voxels prior to feature extraction. Radiomics features were extracted using the pyRadiomics open‐source Python library^53^ (v2.2.0). A total of 103 features were extracted. These consisted of 13 morphology (shape) features, 17 intensity‐histogram (first‐order) features, and 73 textural (Haralick) features. The full list of features and the settings used for pyRadiomics can be found in Table S5. The shape‐related features are not affected by denoising and therefore were excluded from feature reproducibility analysis, resulting in 90 included features. All 103 features were used to derive the 4‐year pre‐treatment survival prediction model and time‐to‐event survival analysis.

## 3 RESULTS

**FIGURE 2:**
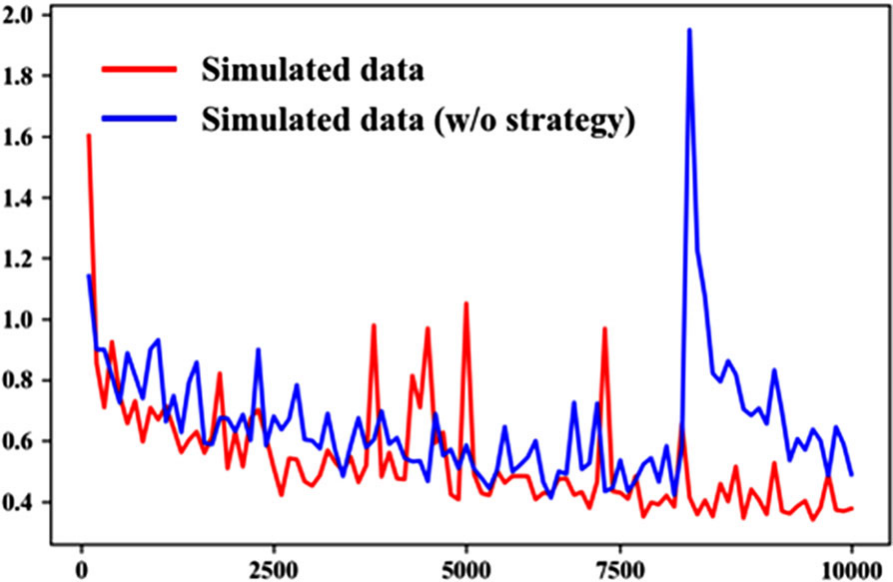
Generator loss over time for cycle GAN training runs with and without slice‐pairing strategy.

Training the cycle GAN from simulated and real data took 96 and 72 h, respectively. The loss of the generator during training is shown in Figure 2. We choose to plot steps rather than epochs in loss curves because, plotting epochs would make it harder to observe the turbulence of model training and the faster convergence of the slice‐paired training strategy. Moreover, the size of the training dataset in the simulated dataset is different to the real dataset, and plotting epochs would be an unfair comparison.

### 3.1 Reproducibility of radiomic features on simulated paired data

In the phantoms’ dataset, the RMSEs of denoised versus high‐dose CT scans using the cycle GAN trained on simulated data and the cycle GAN trained on real data were 0.0187 and 0.0226, respectively, compared with 0.0231 in the original low‐dose CTs. The encoder–decoder network and CGAN trained on simulated data achieved RMSEs of 0.0182 and 0.0140, respectively, in same dataset. Based on visual inspection, we did not detect any image artifacts introduced during the cycle GAN‐based denoising.

An example of an original, noisy and denoised CT scan is shown in Figure 4. We reuse results of CGAN and EDN from^13^ for better comparison with the cycle GAN (corresponding figure for high noise image is Figure S3). In addition, Table 3 shows the RMSE, perceptual loss, signal‐to‐noise ratio (SNR), and ratio of radiomic features with poor (CCC<0.65), medium (0.65≤CCC<0.85), and good (CCC ≥0.85) reproducibility.^49^ The full result of CCC for every feature at different training epochs can be found in Table S6–7.

**TABLE 3:** Summary of RMSE, perceptual loss, and distribution of CCCs of radiomic features based on denoising simulated datasets

Distribution							
Models	RMSE	Perceptual loss	SNR (dB)	CCCs < 0.65 (%)	0.65 ≤ CCCs < 0.85 (%)	CCCs ≥ 0.85 (%)	95%CI of CCC
Low‐noise images							
Without denoising	0.0225	0.0706	18.3	10	22	68	(0.833, 0.901)
Encoder‐decoder	0.0173	0.0427	19.6	0	19	81	(0.901, 0.935)^a^
CGAN	0.0143	0.0290	21.0	3	17	80	(0.905, 0.939)^a^
Cycle GAN	0.0170	0.0216	24.6	0	16	84	(0.916, 0.949)
Cycle GAN (w/o slice pairing)	0.0167	0.0258	20.8	1	13	86	(0.924, 0.957)
Cycle GAN (real data)	0.0229	0.0531	15.7	6	52	42	(0.788, 0.834)
High‐noise images							
Without denoising	0.0237	0.0781	6.1	36	23	41	(0.617, 0.745)
Encoder‐decoder	0.0175	0.0443	19.3	4	16	80	(0.901, 0.935)^a^
CGAN	0.0146	0.0305	20.8	0	16	84	(0.905, 0.939)^a^
Cycle GAN	0.0181	0.0245	20.3	0	14	86	(0.928, 0.954)
Cycle GAN (w/o slice pairing)	0.0188	0.0256	19.4	3	12	84	(0.917, 0.953)
Cycle GAN (real data)	0.0230	0.0501	15.4	4	54	42	(0.779, 0.827)

^a^ Results reproduced from.^13^

**FIGURE 3:**
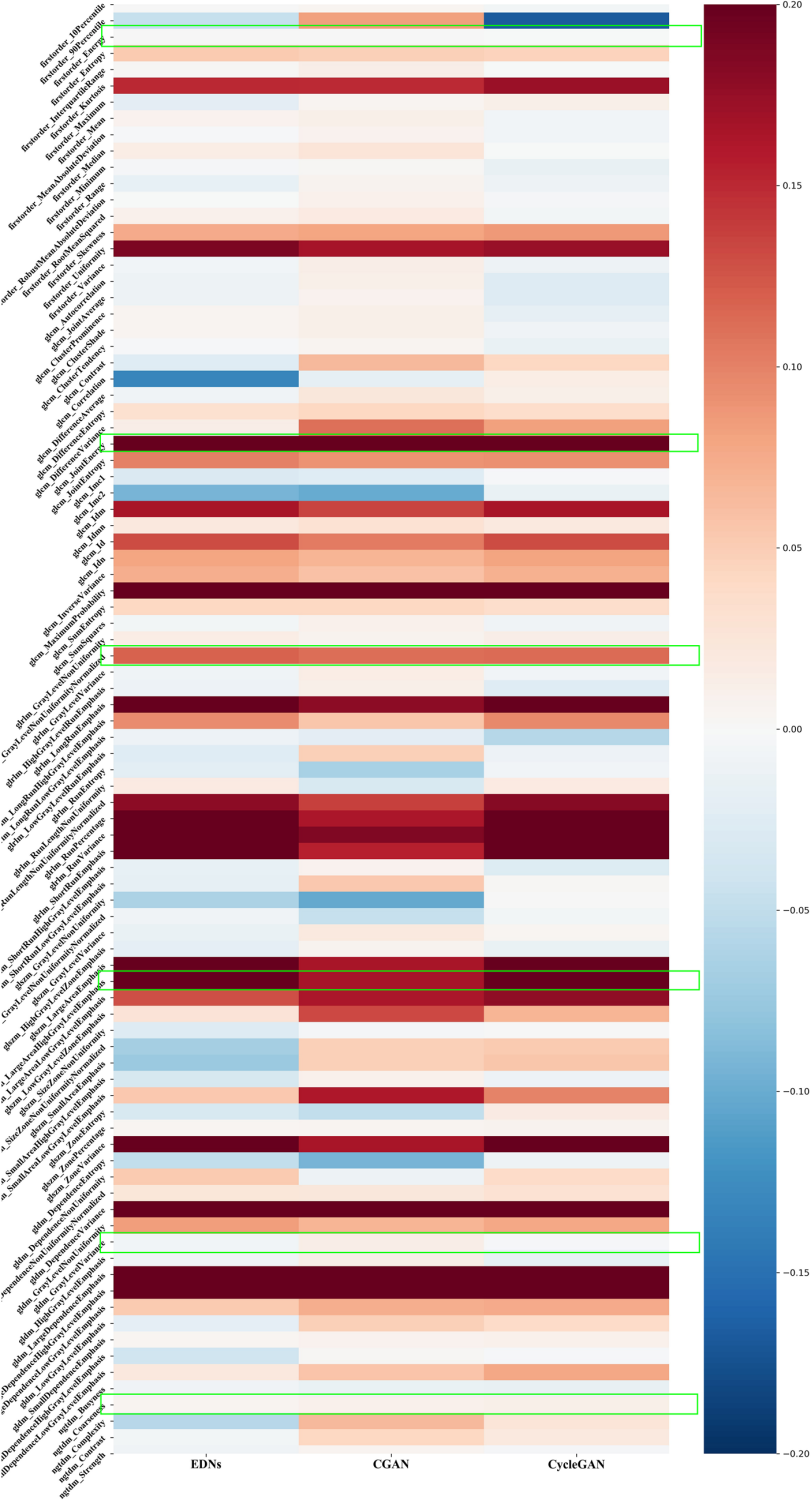
A heatmap of radiomics improvement from denoised low‐noise images, results on EDN and CGAN are reproduced from^13^

As shown in Table 3, the RMSE and perceptual loss of low‐noise and high‐noise images (before denoising) are 0.0225/0.0706 and 0.0237/0.0781, respectively. The cycle GAN trained on simulated data reduced the RMSE and perceptual loss to 0.170/0.216 and 0.0181/0.0245 for low‐noise and high‐noise images; the cycle GAN trained on real data increased RMSE and perceptual loss to 0.0229/0.0531 for low‐noise images and decreased RMSE and perceptual loss to 0.0230/0.0501 for high‐noise images. The cycle GAN trained on simulated data resulted in higher RMSE than the CGAN but lower perceptual loss and outperformed the encoder–encoder network in both metrics. The cycle GAN trained on real data has a worse performance in denoising simulated noisy images compared to other networks. The mean CCCs for cycle GAN trained on simulated data denoised images improved from 0.87 (95% CI, [0.833,0.901]) and 0.68 (95% CI, [0.617,0.745]) to 0.93 (95% CI, [0.916, 0.949]) and 0.94 (95% CI, [0.928,0.954]) for low‐noise images and high‐noise images, respectively (Wilcoxon rank‐sum test for the CCC from noisy images and denoised images, *p*‐value<0.01 for both experiments). The mean CCCs of low noise images denoised with the cycle GAN trained on real data decreased to 0.81 (95% CI, [0.788,0.834]) and the mean CCCs of denoised high noise images increased to 0.80 (95% CI, [0.779,0.827]) (Wilcoxon rank‐sum test comparing CCC of noisy images and denoised images: *p*‐value<0.01 for both experiments). A heatmap of radiomics improvement from denoised low‐noise images by comparing with original noisy images is shown in Figure 3.

In contrast, EDN and CGANs were able to improve the mean CCC of radiomic features to 0.92 (95%CI, [0.909,0.936]) for low and high‐noise images. The cumulative distribution function (CDF) of CCCs for different models when trained for 100 epochs is shown in Figure 5a,b. The cycle GAN trained on real data did not manage to improve radiomics features’ reproducibility on simulated noisy images. However, it still achieved a significant improvement in the reproducibility of radiomics features of simulated high noise images.

The second investigation of the simulation study was the effect of different training epochs to radiomics reproducibility. The CDF of CCCs for cycle GAN trained at 25, 50, 75, and 100 epochs are shown in Figure S4a,b. Summary of RMSE, perceptual loss, and CCCs of cycle GAN trained at different epochs can be found in Table S8. We compared the CCC distributions of radiomic features calculated on images denoised from high‐noise images with those of images denoised from low‐noise images using the Wilcoxon rank‐sum test resulting in a *p*‐value of 0.94. The results show that a cycle GAN trained to denoise high‐noise images can be applied to denoise images with different levels of noise and achieve similar results to a CGAN and EDN‐based denoiser.^13^ Moreover, we compared the CCC distributions from cycle GAN with CGAN and EDN by using the Wilcoxon rank‐sum test which resulted in *p*‐values of 0.73 and 0.07, respectively. The results show that a cycle GAN achieved similar results to CGAN and EDN, and that in some cases, Cycle Gan even received better results.

### 3.2 Ablation study for the training strategy

**FIGURE 4:**
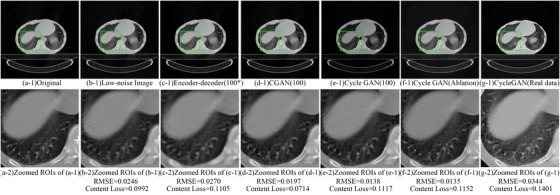
Example of low‐dose CT denoising. (a‐1) The original full dose CT image; (b‐1) Low‐noise image; (c‐1) image denoised by EDN (*Training at 100 epochs); (d‐1) image denoised by CGAN; (e‐1) image denoised by a cycle GAN; (f‐1) image denoised by a cycle GAN (ablation study); (g‐1) image denoised by cycle GAN trained on real data; (a‐2) to (g‐2) zoomed ROIs for (a‐1) to (g‐1).

An example of denoised images from cycle GAN ablation study can be found in Figure 4f‐1,f‐2.

**FIGURE 5:**
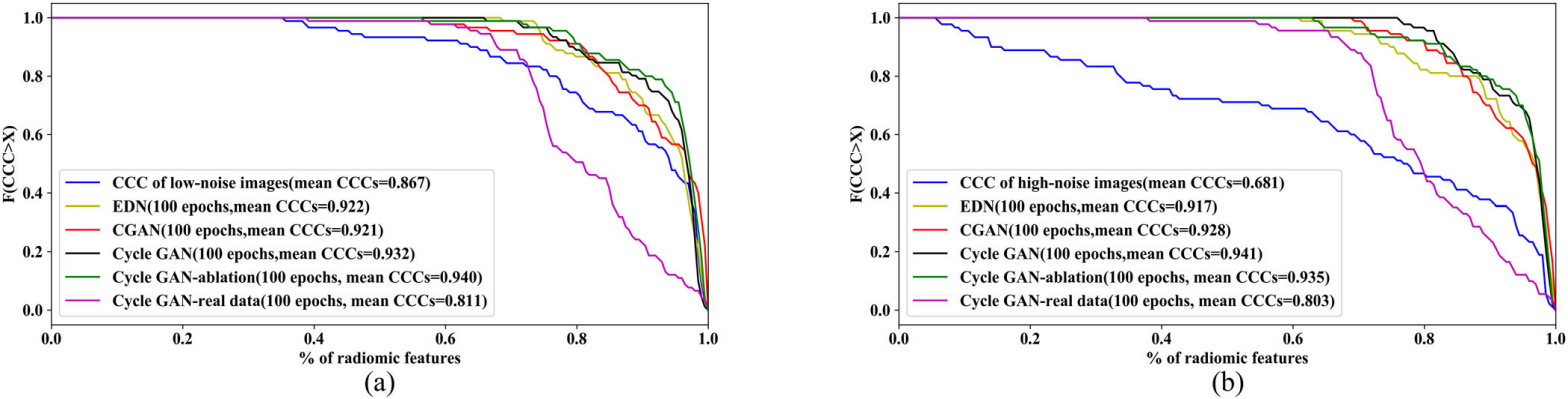
CDF of CCC of radiomic features denoised with different models. (a) Low‐noise images; (b) high‐noise images.

Table 3 and Table S9 show the RMSE, perceptual loss, and ratio of poor, medium, and good reproducibility radiomic features about ablation study of cycle GAN. The cycle GAN trained without our training strategy can also reduce the RMSE and perceptual loss of low‐noise and high‐noise images to 0.0167/0.0258 and 0.0188/0.0256, respectively. Moreover, it can increase the average CCC to 0.94 (95%CI, [0.924,0.957]) and 0.93 (95%CI, [0.917,0.953]) for low‐ and high‐noise images, respectively. The CDF of CCCs for ablation study when trained for 100 epochs is shown in Figure 5a,b, and the differences among epochs can be found in Figure S4c,d. The distribution of CCCs from ablation study trained at 100 epochs was compared with results from a network trained with training strategy, and we found no signification differences (Wilcoxon rank‐sum test, *p*‐value = 0.13). Figure 2 shows that training the cycle GAN with the training strategy might speed up convergence slightly. On the other hand, without the training strategy, the generator's loss function increases beyond 60,000 steps. Finally, the cycle GAN trained with our training strategy led to significantly higher CCCs when trained for only 25 epochs (Wilcoxon rank‐sum test, *p*‐value < 0.01), as shown comparing Figure S4a–c and Figure 4b–d.

### 3.3 Reproducibility on real data

**FIGURE 6:**
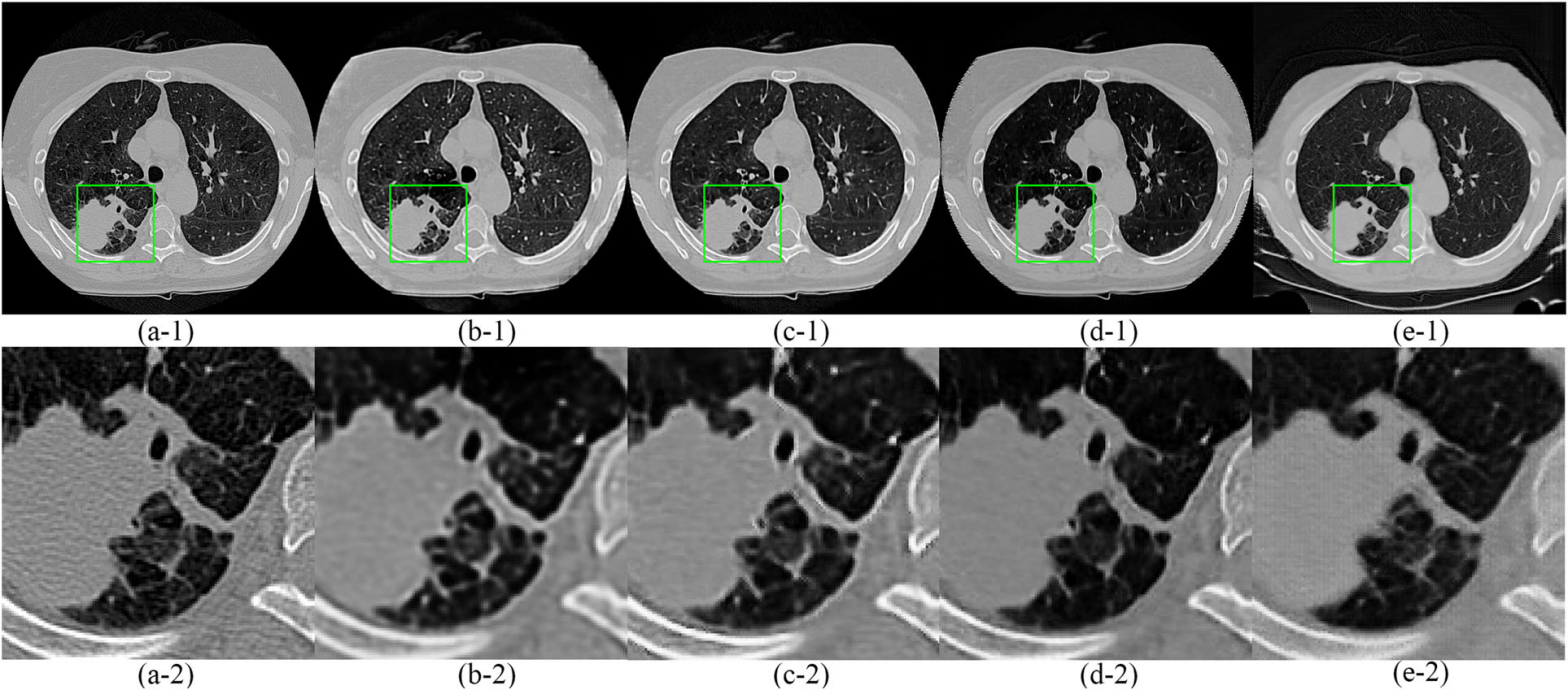
Example of RIDER denoising. (a‐1) One original image from RIDER; (b‐1) image denoised by EDN (Training at 100 epochs); (c‐1) image denoised by CGAN (training at 100 epochs); (d‐1) image denoised by cycle GAN trained on simulated data (100 epochs); (e‐1) image denoised by cycle GAN trained on real data (100 epochs); (a‐2) to (e‐2) zoomed ROIs for (a‐1) to (e‐1).

**FIGURE 7:**
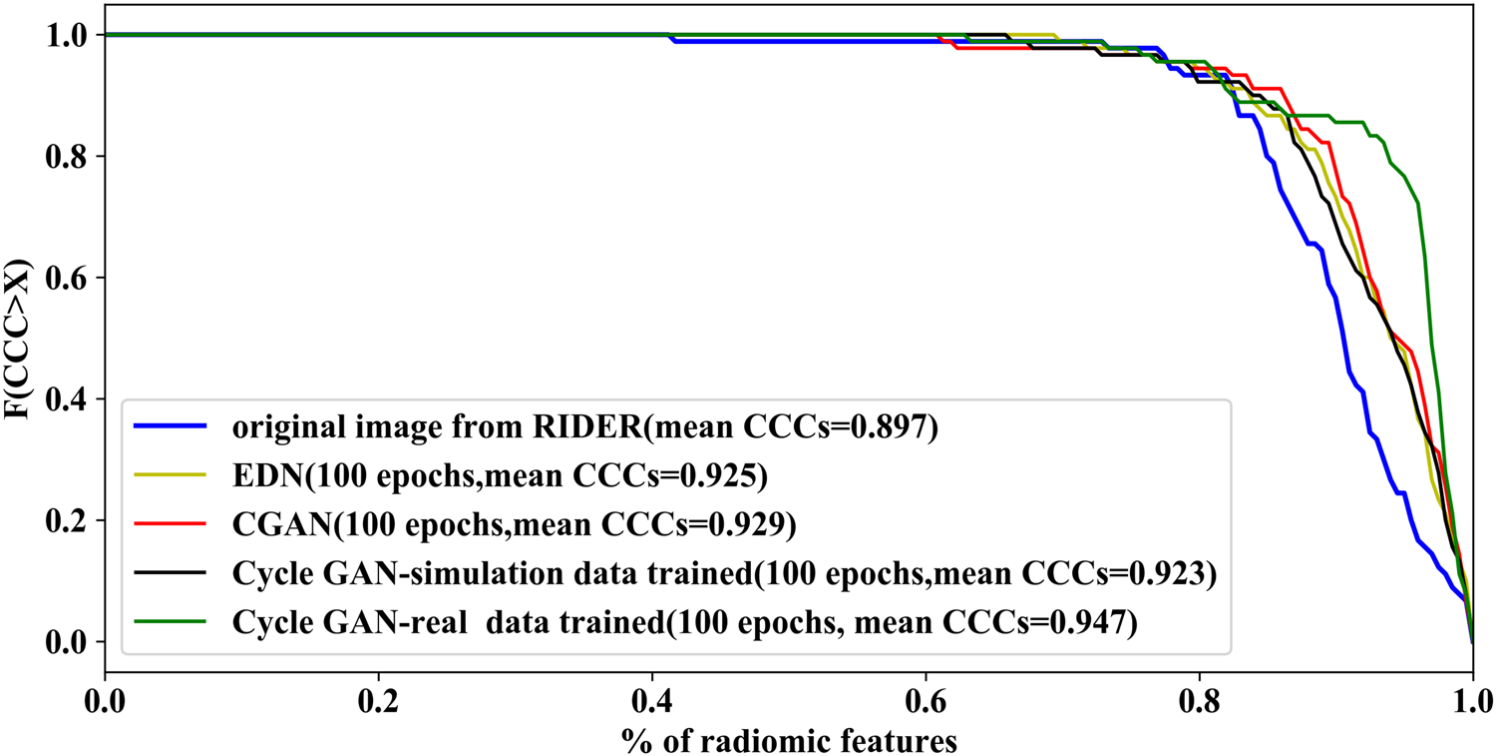
CDF of CCCs and for denoised CT scans in the RIDER dataset.

We now focus on the impact of denoising on the reproducibility of radiomic features in same day repeat low‐dose CT scans (RIDER dataset). An example of an original image and its denoised counterparts denoised using a CGAN, an EDN, and the cycle GANs trained on simulated and real data are shown in Figure 6. Figure 7 shows the CDF of the CCCs for the radiomic features extracted from the original and denoised CT images. The cycle GAN trained on real data outperforms the rest of generative models (Wilcoxon rank‐sum test, *p*‐value < 0.01). On the other hand, the performance of the cycle GAN trained on simulated data is similar to that of the EDN and CGAN (*p*‐value = 0.87 and 0.40, respectively).

### 3.4 Survival prediction on real data

An example of an original NSCLC Radiogenomics image, and its denoised counterparts based on CGAN, EDN, and cycle GANs trained from simulated and real data can be found in Figure S5.

**FIGURE 8:**
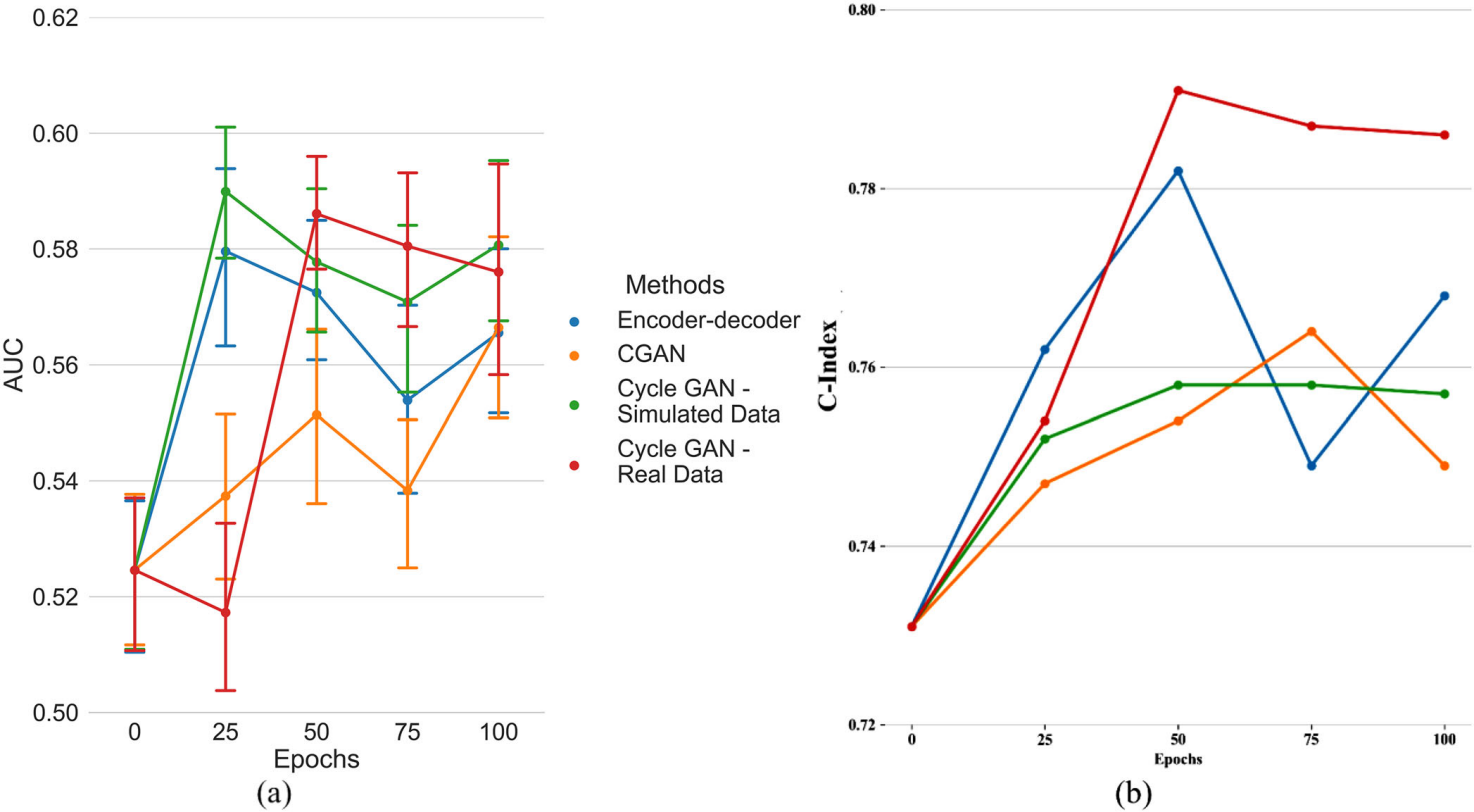
Results of 4‐year pretreatment survival prediction (a) and time‐to‐event survival analysis; (b) C‐index of survival analysis.

Figure 8a illustrates the results of the of 4‐year pre‐treatment survival prediction experiment showing the AUC for each generative model across different number of epochs. We achieved an AUC for survival prediction based on radiomics extracted from the original NSCLC Radiogenomics dataset of 0.52 (95%CI, [0.511,0.538]) at 100 epochs. Denoising the CT scans using a CGAN or an EDN led to models with an increased AUC of 0.57 (95%CI, [0.551, 0.580]) (at 100 epochs) as shown in.^39^ The cycle GANs trained on simulated and real data resulted in a higher mean AUC of around 0.58 (95%CI, [0.576,0.596]), but the difference between models was not statistically significant (Student's t‐test, all *p*‐values > 0.10). Figure 8b illustrates the results of the time‐to‐event survival analysis experiment showing the C‐index for each generative model across different numbers of epochs. EDN, CGAN, and the cycle GAN trained on simulated data improved C‐index of survival analysis from 0.73 to around 0.76, while the cycle GAN trained on real data improved the C‐index to 0.78.

To interpret the improvement of AUC in 4‐year survival prediction tasks, we used an RBF kernel‐based SVM Recursive Feature Elimination algorithm^54^ to assess the importance of features in the prediction model. Table 4 shows the top eight most important features in the models trained on the radiomic features from the original images and those from denoised images (the table with all features can be found in Tables S10–S13). Six features appeared in all four models (highlighted in green in Figure 3). These features of CCC improved by denoisers, most of them improved significantly, which might explain how denoising can improve the AUC of survival prediction models.

**TABLE 4:** Top eight most important features in the survival prediction model trained on noisy images and images denoised using different generative models

Rank	Original images	Denoised with EDN	Denoised with CGAN	Denoised with cycle GAN
1	glszm_LargeArea LowGrayLevelEmphasis	glszm_LargeArea LowGrayLevelEmphasis	glszm_LargeArea LowGrayLevelEmphasis	glrlm_GrayLevel NonUniformityNormalized
2	ngtdm_Coarseness	gldm_GrayLevelVariance	glrlm_GrayLevel NonUniformityNormalized	glszm_LargeArea LowGrayLevelEmphasis
3	gldm_GrayLevelVariance	glszm_LargeArea LowGrayLevelEmphasis	gldm_GrayLevelVariance	gldm_GrayLevelVariance
4	firstorder_Energy	gldm_LargeDependence HighGrayLevelEmphasis	firstorder_Energy	firstorder_Energy
5	shape_MinorAxisLength	gldm_GrayLevel NonUniformity	gldm_GrayLevel NonUniformity	shape_MinorAxisLength
6	glrlm_GrayLevel NonUniformityNormalized	firstorder_Energy	ngtdm_Coarseness	ngtdm_Coarseness
7	glszm_LargeArea HighGrayLevelEmphasis	glcm_JointEntropy	glcm_JointEntropy	glcm_JointEntropy
8	glcm_JointEntropy	ngtdm_Coarseness	shape_MinorAxisLength	glrlm_RunLength NonUniformityNormalized

Shape features, which were previously excluded from denoising analyses, were included as candidate predictors for the survival prediction model. However, as shown in Table 3, there are no shape features among the top eight most important predictors.

## 4 DISCUSSION

The objective of our study was to investigate the potential of cycle GANs for denoising low dose CTs to improve the reproducibility of radiomics features and the performance of radiomics‐based models. For this purpose, we trained two cycle GANs, one with simulated paired data and the other one with real data, to denoise low‐dose CT scans. In order to measure the performance of our denoising models, we ran experiments and compared the results of our method with those of CGANs and EDNs trained on simulated paired data. The results show that both cycle GANs trained on simulated and on real data can improve radiomics’ reproducibility and performance in low‐dose CT and achieve similar results compared to CGANs and EDNs.

**FIGURE 9:**
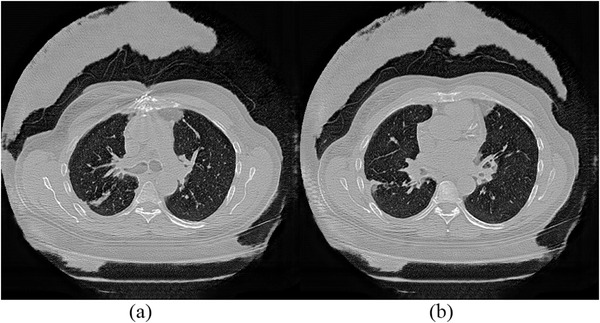
Examples of failed cycle GAN training.

The main advantage of cycle GANs over CGANs and EDNs is that they do not require paired images, which are hard to collect. For CGANs and EDNs, we overcame this issue by generating simulated low‐dose CTs by introducing noise into high‐dose CTs.^13^ However, simulated noise might differ from noise encountered in low‐dose CTs. Hence, being able to train a model on real low‐dose CT scans is a significant advantage. However, training cycle GANs is volatile, especially when the target domain and the source domain differ, as documented elsewhere.^41^, ^55^ Ideally, in order to maximize the chances of success for the training process, training data would be collected from the same scanner, with the same protocol (except radiation exposure), and from the same group of patients for the two domains (low‐ and high‐dose CT). However, such a dataset is not available to us. Hence, we defined selection criteria for the training data so that the source and target image domains kept certain similarities. We chose scanner manufacturer and table height (which determines field of view and the height of human body) based on.^12^ These inclusion criteria were introduced after several failed attempts at training a cycle GAN with the full dataset. Examples of failed training runs are shown in Figure 9. However, trained models retain certain generalizability and can achieve good results across different scanners with different parameter settings as shown in the results (images in the RIDER and NSCLC Radiogenomics datasets were scanned from multiple types of scanners with different protocols).

As shown in Table 1, the Lung 1 dataset differs more in terms of scanning parameters from the RIDER and NSCLC Radiogenomics datasets compared to the LIDC‐IDRI and TCGA‐LUAD datasets. It is therefore possible that the conditional GAN trained on simulated paired TCGA‐LUAD data achieved a similar performance as the cycle GAN trained on real data. Future studies may confirm this hypothesis.

Our ablation study results seem different from research reported elsewhere^43^ which found that a slice‐based training strategy can improve denoising performance. The slice‐paired training strategy we proposed seems to lead to slightly faster convergence as hinted by the loss plot and the models’ results at 25 epochs. However, this strategy did not lead to significant improvement of the networks’ denoising performance at 100 epochs. One possible explanation is that the training strategy cannot make the resulting network a better approximator of the mapping from low‐dose CT domain to high dose. Figure 2 and the comparisons between Figure S4a,c and Figure S4b,d seem to support this view. Another possible hypothesis for this phenomenon is that reproducibility and performance of radiomics may not be so sensitive to the quality of images when the quality reaches a certain threshold. We did not report results of the ablation study for the slice‐paired training strategy when training on real data because the training of the cycle GAN failed to converge multiple times without slice pairing. The failure to converge was probably due to a higher heterogeneity in real data compared to simulated data (simulated data were collected from the same scanners while real data were collected from different scanners). Thus, the slice‐pairing strategy seems to have made the network training more stable in our study.

As mentioned above, cycle GANs achieved a similar performance as CGAN and EDN trained on simulated data, slightly outperforming them in some experiments. The difference in performance might be explained by the differences in the architectures used: the generator in CGAN and the encoder–decoder is a five‐layer network, while there are nine ResNet blocks^56^ (27 convolutional layers) in the cycle GAN's generators. Related articles have hypothesized^22^ that neural networks for ‘low level’ domain adaptation—such as denoising—should be kept shallow, since texture transfer in ‘low level’ domain adaptation is not significant. However, the results in our study seem to show that very deep neural network can also achieve good performance in some ‘low level’ domain adaptation tasks.

Our training cohort population is smaller than the testing cohort population for two main reasons. First, we considered the size of our training sets (ranging from 3144 frames to 4260 frames) was sufficient based on 2D cycle GAN training set examples in the literature, that range between several hundreds to a few thousands.^41^, ^57^ Secondly, CCC of radiomics is sensitive to the number of subjects used. Moving more subjects (images) from testing datasets to training datasets would decrease the reliability of radiomics features’ CCC calculations.

As shown in Figure 6, the cycle GAN trained on simulated data (Figure 6d) seems to have a better denoising performance in some cases in terms of tissue enhancement and intensity smoothing on homogeneous regions compared with the model trained on real data (Figure 6e). Of course, it might just be that among the tens of thousands of CT images in the experiment, this is one where the cycle GAN trained on simulated data fared better than its counterpart trained on real data. In addition, it might be that the data distribution (as well as the noise) in the simulated training data is more homogeneous than the real data, and this might lead to more appealing visual results, but statistical metrics point at a consistently superior performance by the model trained on real data.

One potential limitation of our study is the low AUCs achieved by the models for pretreatment survival prediction for lung cancer based on radiomic features. However, these are in line with results reported elsewhere. For example, Isensee et al.^58^ reported an accuracy of 52.6% based on the BraTS 2017 dataset^59^ for brain tumors using radiomics; Choi et al.^60^ reported an integrated AUC (iAUC) of 0.620 (95% CI: 0.501–0.756]) using the TCGA/TCIA dataset and random survival forest to derive a prediction model. Finally, Bae et al.^61^ reported an iAUC of 0.590 (95% CI: 0.502, 0.689) for overall survival prediction in glioblastoma using MRI radiomic features.

Our study suffered from a few other limitations. First, there were important differences between the populations in different training datasets (LIDC‐IDRI and TCGA‐LUAD). For example, patients in TCGA‐LUAD were thinner than patients in LIDC‐IDRI, as shown in Figure S4. Hence, the cycle GAN trained on these datasets learnt to not only denoise the images, but make the patients thinner as illustrated in Figure 6e‐1. Fortunately, the ROIs of this study are located in the lung, and the volume of patients’ lung in two domains are similar. Therefore, there was no significant size shift in the ROIs. Second, due to the differences of the CT bed in LIDC‐IDRI and TCGA‐LUAD, the cycle GAN also transforms bottom part of the image as shown in Figure 6e‐1. Third, the cycle GAN trained on real data performed relatively poorly on simulated noisy images in terms of improving the reproducibility of radiomic features. One of the potential reasons is the domain distribution gap between real data and simulated data. The variations of scanners, patient cohorts, reconstruction algorithms in real training dataset may reduce the network's denoising performance in the simulated dataset.^62^ Moreover, we believe that the good performance in real data is more important than the performance in simulated data, since it is more representative of real applications. Fourth, one of the assumptions of our slice‐paired training strategy is that the first slice of a low‐dose CT scan will have higher similarity with the first slice of a high‐dose CT scan, is not automatically true. The similarity of the first slice of a CT scan depends on a lot of factors such as the patient position, section of the body scanned, and so forth. These factors were ignored in this paper. Fifth, as we mentioned in section 2.4, no early stopping of training was adopted in this study. However, as shown in Figures 2, 5 and Figure S4, we cannot witness the improvements of the model's performance during training. This may mean that the generator of the cycle GAN does not learn the real data distribution, since the loss function fluctuated in all training steps (50,000 steps in our case). Therefore, early stopping techniques and AutoML‐based hyperparameter selection^63^ seem like promising topics for further research. Sixth, in this study, the trained models were only tested in two applications: improving radiomics reproducibility in same‐day repeat low‐dose CTs and radiomics performance survival prediction. More experiments to better understand the relationship between denoising and radiomics performance are needed. Seventh, radiation dose is not the only source of lack of reproducibility of radiomics’ features,^64^ and in some cases, it might not be the most relevant.^12^ Therefore, a denoising model might not solve all the reproducibility issues of radiomic features and other measures will need to be put in place to address other sources of inconsistency (slice thickness, reconstruction parameters, contrast enhancement, etc.). Our GANs were trained on datasets collected from different scanners with different scanning parameters, and images were reconstructed using different software and kernels. This might lead to more robust models, but at the same time, we cannot guarantee that our trained GAN does not introduce new inconsistencies to radiomics. Moreover, there are some deep learning‐based methods for extracting radiomics features, usually referred to as ‘deep radiomics’.^65^, ^66^ However, there are limited studies focusing on extracting features from low‐dose CT and, to the best of our knowledge, no study focused on improving deep radiomics reproducibility or performance in low‐dose CT. Studies focusing on assessing deep radiomics’ reproducibility and performance in low‐dose CT would be of interest. Eighth, we did not compare the performance of the cycle GAN with non‐AI commercial low‐dose CT reconstruction algorithms, such as model‐based iterative reconstruction (MBIR)^67^). Such a comparison would be of interest, but we could not perform it in our study due to the absence of sinograms (which are required to use reconstruction algorithms) in the datasets used. Finally, due to the absence of a structure similarity term in our cycle GAN's cost function, some images develop distortions in microstructures. Therefore, further adjustments on cost function and network architecture should be assessed in the future.

## 5 CONCLUSIONS

In this study, we investigate the potential of denoising low‐dose CT using cycle GANs to improve the reproducibility of radiomics features and the performance of radiomics‐based prediction models. We trained two cycle GANs: using paired simulated low‐dose CTs and unpaired real low‐ and high‐dose CT images. To accelerate convergence, we introduced a slice‐paired training strategy.

The results of our experiments show that a cycle GAN trained to denoise low‐dose CT scans from unpaired low‐ and high‐dose CT scans can improve the reproducibility of radiomic features in simulated low dose CTs and same‐day repeat low dose CTs. In addition, we showed that radiomics based pretreatment survival prediction models trained on low‐dose CT scans denoised with said cycle GAN can achieve better performance. The improvement in reproducibility and prediction model performance are comparable to those achieved with CGANs and encoder–decoder networks trained on simulated paired data. Cycle GANs may have a better future potential because they do not need paired data, but they are burdened by the volatility of the treatment process, which limits their applicability. More research is needed to make cycle GAN training more robust, for them to be able to be trained on a more diverse dataset.

## CONFLICT OF INTEREST

The authors declare no conflicts of interest.

## AUTHOR CONTRIBUTIONS

Study conception and design: JC and IB. Data acquisition and analysis: JC and LW. Manuscript writing and revision: JC, AD, LW and IB. Supervision: AD, LW and IB.

## Supporting information

Supplementary Materials
