# Supplementary material for: Improving reproducibility and performance of radiomics in low‐dose CT using cycle GANs
Source: J Appl Clin Med Phys. 2022 Jul 30;23(10):e13739. doi: 10.1002/acm2.13739 (PMC9588275; doi:10.1002/acm2.13739)
Supplement: Supplementary file 1 — Supplementary Materials [file ACM2-23-e13739-s001.pdf]

Supplementary Materials

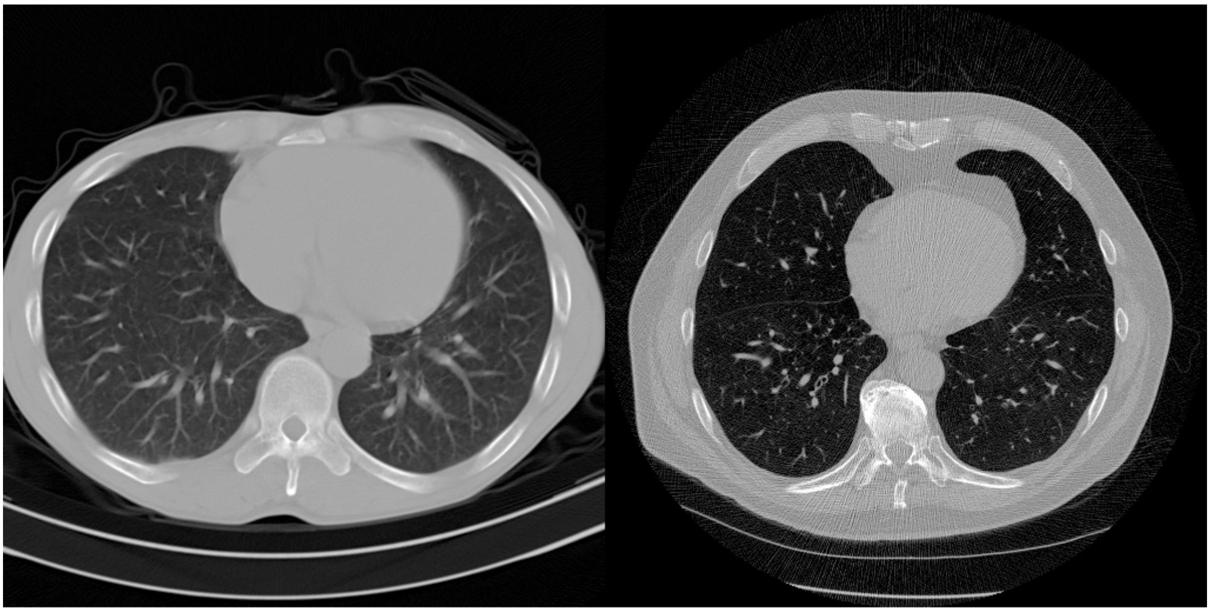

Supplementary Figure 1. An example image of selected samples from TCGA-LUAD and LIDC-IDRI. (a) an example for high dose CT image from TCGA-LUAD; (b) an example for low dose CT image from LIDC-IDRI.

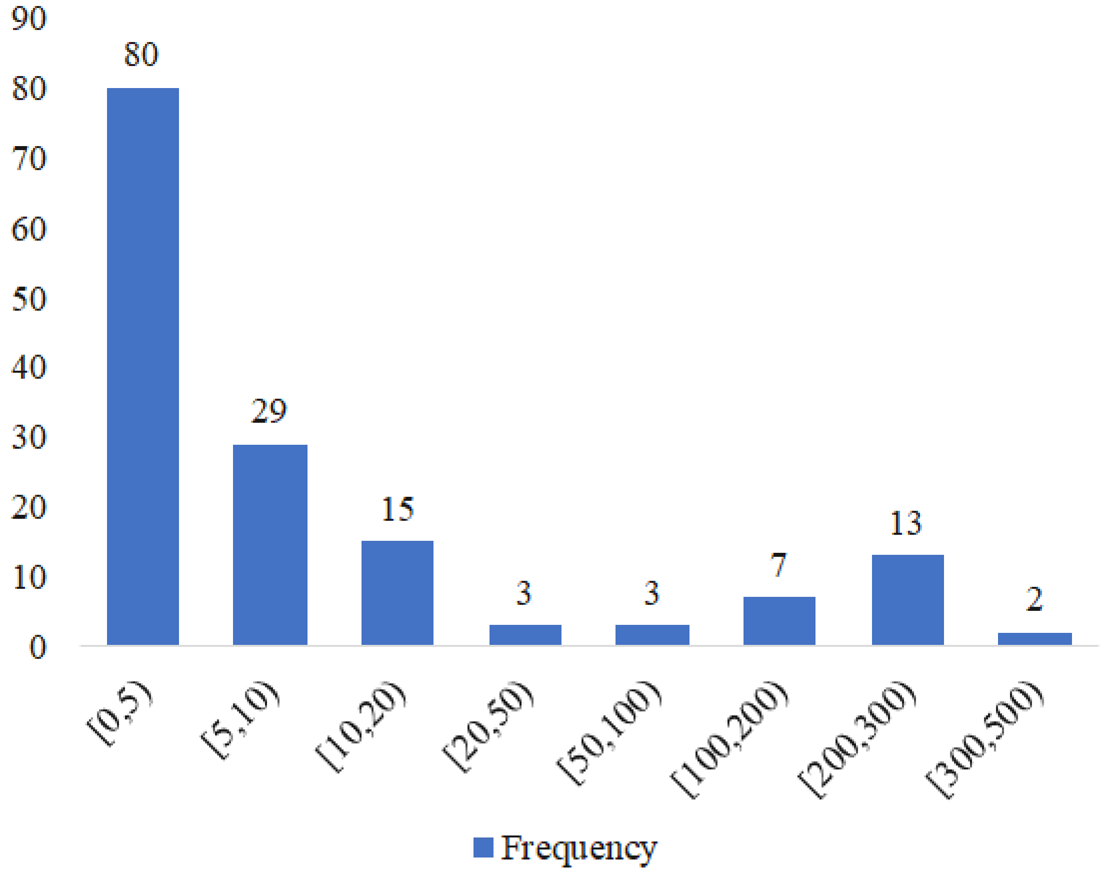

Supplementary Figure 2. The distribution of radiation exposure for selected samples in the NSCLC Radiogenomics

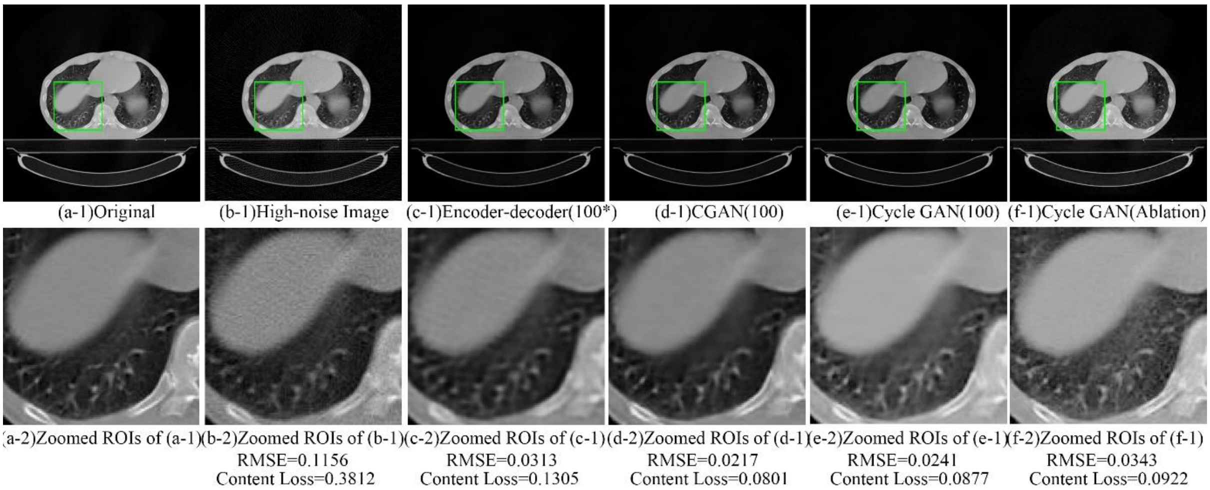

Supplementary Figure 3. Example of low dose CT denoising. (a-1) The original full dose CT image; (b-1) high-noise image; (c-1) Image denoised by encoder-decoder network (*Training at 100 epochs); (d-1) Image denoised by CGAN; (e-1) Image denoised by cycle GAN; (f-1) Image denoised by cycle GAN (ablation study); (a-2) to (f-2) Zoomed ROIs for (a-1) to (f-1).

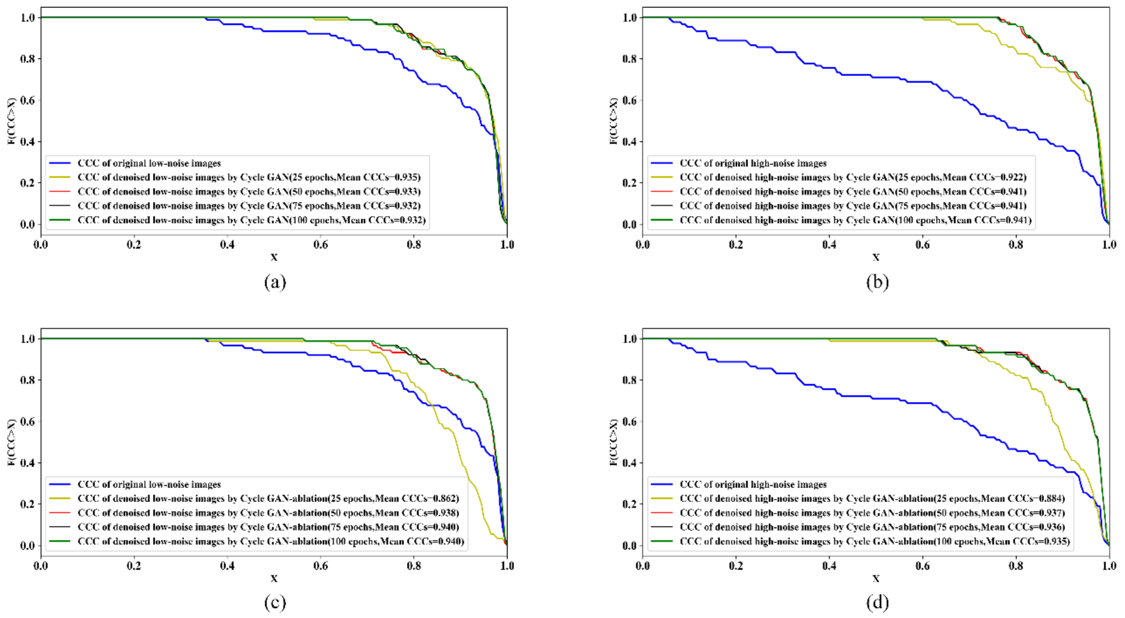

Supplementary Figure 4. CDF of CCC by Using Cycle GAN. (a) CDF of CCCs based on denoised low-noise by using Cycle GAN trained for different numbers of epochs; (b) CDF of CCCs based on denoised high-noise by using Cycle GAN trained for different numbers of epochs; (c) CDF of CCCs based on denoised low-noise by using Cycle GAN trained without strategy for different numbers of epochs; (d) CDF of CCCs based on denoised high-noise by using Cycle GAN trained without strategy for different numbers of epochs.

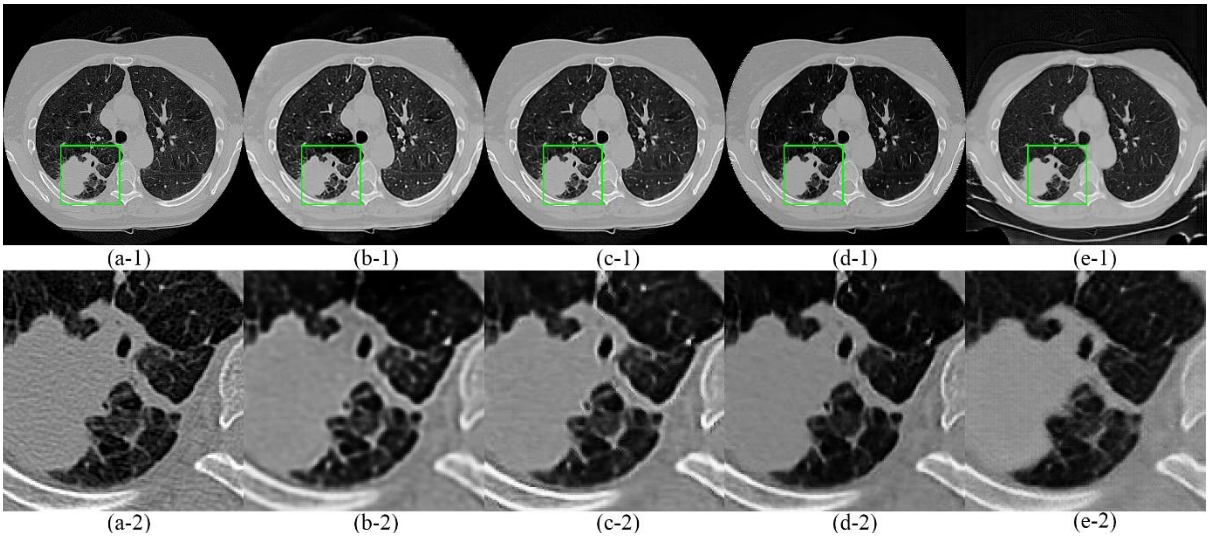

Supplementary Figure 5. Example of RIDER denoising. (a-1) One original image from RIDER; (b-1) Image denoised by encoder-decoder network (Training at 100 epochs); (c-1) Image denoised by CGAN (Training at 100 epochs); (d-1) Image denoised by simulation data trained Cycle GAN (Training at 100 epochs); (e-1) Image denoised by real data trained Cycle GAN (Training at 100 epochs); (a-2) to (e-2) Zoomed ROIs for (a-1) to (e-1).

Supplementary Table 1. Index of available patients for experiments in LUNG 1

Training Samples	LUNG1-001	LUNG1-029	LUNG1-032	LUNG1-051	LUNG1-072	LUNG1-098
	LUNG1-100	LUNG1-105	LUNG1-109	LUNG1-115	LUNG1-116	LUNG1-119
	LUNG1-120	LUNG1-121	LUNG1-122	LUNG1-124	LUNG1-126	LUNG1-127
	LUNG1-128	LUNG1-130	LUNG1-131	LUNG1-132	LUNG1-133	LUNG1-134
	LUNG1-139	LUNG1-141	LUNG1-142	LUNG1-145	LUNG1-147	LUNG1-148
	LUNG1-151	LUNG1-152	LUNG1-156	LUNG1-157	LUNG1-160	LUNG1-161
	LUNG1-162	LUNG1-163	LUNG1-165	LUNG1-169		
Testing Samples	LUNG1-171	LUNG1-172	LUNG1-174	LUNG1-176	LUNG1-178	LUNG1-179
	LUNG1-189	LUNG1-191	LUNG1-192	LUNG1-195	LUNG1-196	LUNG1-197
	LUNG1-199	LUNG1-205	LUNG1-206	LUNG1-210	LUNG1-211	LUNG1-212
	LUNG1-213	LUNG1-214	LUNG1-215	LUNG1-216	LUNG1-217	LUNG1-218
	LUNG1-220	LUNG1-221	LUNG1-222	LUNG1-223	LUNG1-224	LUNG1-225
	LUNG1-226	LUNG1-227	LUNG1-229	LUNG1-231	LUNG1-233	LUNG1-234
	LUNG1-235	LUNG1-236	LUNG1-237	LUNG1-239	LUNG1-243	LUNG1-244
	LUNG1-245	LUNG1-247	LUNG1-249	LUNG1-252	LUNG1-253	LUNG1-255
	LUNG1-256	LUNG1-257	LUNG1-259	LUNG1-260	LUNG1-262	LUNG1-263
	LUNG1-264	LUNG1-266	LUNG1-267	LUNG1-268	LUNG1-269	LUNG1-270
	LUNG1-271	LUNG1-272	LUNG1-273	LUNG1-274	LUNG1-275	LUNG1-276
	LUNG1-277	LUNG1-278	LUNG1-280	LUNG1-282	LUNG1-283	LUNG1-284
	LUNG1-285	LUNG1-287	LUNG1-288	LUNG1-289	LUNG1-290	LUNG1-293
	LUNG1-294	LUNG1-295	LUNG1-296	LUNG1-297	LUNG1-298	LUNG1-299
	LUNG1-300	LUNG1-303	LUNG1-304	LUNG1-305	LUNG1-306	LUNG1-307
	LUNG1-309	LUNG1-310	LUNG1-311	LUNG1-313	LUNG1-315	LUNG1-317
	LUNG1-318	LUNG1-320	LUNG1-321	LUNG1-323	LUNG1-326	LUNG1-328
	LUNG1-331	LUNG1-332	LUNG1-334	LUNG1-335	LUNG1-337	LUNG1-339
	LUNG1-340	LUNG1-341	LUNG1-342	LUNG1-343	LUNG1-345	LUNG1-347
	LUNG1-349	LUNG1-353	LUNG1-354			

Supplementary Table 2. Index of available patients for real data Cycle GAN training in LIDC-IDRI (Low Dose CT Domain)

Training Samples	LIDC-IDRI-0218	LIDC-IDRI-0306	LIDC-IDRI-0336	LIDC-IDRI-0394
	LIDC-IDRI-0430	LIDC-IDRI-0603	LIDC-IDRI-0604	LIDC-IDRI-0780
	LIDC-IDRI-0854	LIDC-IDRI-0862	LIDC-IDRI-0903	LIDC-IDRI-0953

Supplementary Table 3. Index of available patients for real data Cycle GAN training in TCGA-LUAD (High Dose CT Domain)

Training Samples	TCGA-17-Z017	TCGA-17-Z019	TCGA-17-Z021	TCGA-17-Z027
	TCGA-17-Z029	TCGA-17-Z034	TCGA-17-Z050	TCGA-17-Z051
	TCGA-17-Z053	TCGA-17-Z054	TCGA-17-Z058	TCGA-17-Z059
	TCGA-38-4628	TCGA-50-6591		

Supplementary Table 4. Index of available patients for pre-treatment survival predication in NSCLC Radiogenomics

R01-001	R01-035	R01-062	R01-086	R01-108	R01-133
R01-002	R01-037	R01-063	R01-088	R01-110	R01-134
R01-003	R01-039	R01-064	R01-090	R01-111	R01-135
R01-004	R01-040	R01-066	R01-091	R01-112	R01-136
R01-005	R01-041	R01-067	R01-092	R01-113	R01-138
R01-006	R01-042	R01-068	R01-093	R01-114	R01-139
R01-007	R01-043	R01-070	R01-094	R01-115	R01-141
R01-013	R01-045	R01-071	R01-095	R01-116	R01-142
R01-017	R01-046	R01-072	R01-096	R01-117	R01-144
R01-018	R01-047	R01-073	R01-097	R01-118	R01-145
R01-019	R01-048	R01-074	R01-098	R01-119	R01-146
R01-021	R01-049	R01-075	R01-100	R01-120	
	R01-050	R01-077	R01-101	R01-121	
R01-025	R01-051	R01-079	R01-102	R01-122	
R01-029	R01-052	R01-081	R01-103	R01-123	
R01-030	R01-055	R01-082	R01-104	R01-124	
R01-031	R01-056	R01-083	R01-105	R01-125	
R01-033	R01-060	R01-084	R01-106	R01-128	
R01-034	R01-061	R01-085	R01-107	R01-129	

Supplementary Table 5. Radiomics features calculated by using pyradiomics

	Index	Features	Index	Features
G1[1]	1	shape_Elongation	8	shape_Maximum3DDiameter
	2	shape_Flatness	9	shape_MeshVolume
	3	shape_LeastAxisLength	10	shape_MinorAxisLength
	4	shape_MajorAxisLength	11	shape_Sphericity
	5	shape_Maximum2DDiameterColumn	12	shape_SurfaceArea
	6	shape_Maximum2DDiameterRow	13	shape_SurfaceVolumeRatio
	7	shape_Maximum2DDiameterSlice		
	Index	Features	Index	Features
G2[2]	1	firstorder_10Percentile	46	glrlm_LongRunLowGrayLevelEmphasis
	2	firstorder_90Percentile	47	glrlm_LowGrayLevelRunEmphasis
	3	firstorder_Energy	48	glrlm_RunEntropy
	4	firstorder_Entropy	49	glrlm_RunLengthNonUniformity
	5	firstorder_InterquartileRange	50	glrlm_RunLengthNonUniformityNormalized
	6	firstorder_Kurtosis	51	glrlm_RunPercentage
	7	firstorder_Maximum	52	glrlm_RunVariance
	8	firstorder_Mean	53	glrlm_ShortRunEmphasis
	9	firstorder_MeanAbsoluteDeviation	54	glrlm_ShortRunHighGrayLevelEmphasis
	10	firstorder_Median	55	glrlm_ShortRunLowGrayLevelEmphasis
	11	firstorder_Minimum	56	glszm_GrayLevelNonUniformity
	12	firstorder_Range	57	glszm_GrayLevelNonUniformityNormalized
	13	firstorder_RobustMeanAbsoluteDeviation	58	glszm_GrayLevelVariance
	14	firstorder_RootMeanSquared	59	glszm_HighGrayLevelZoneEmphasis
	15	firstorder_Skewness	60	glszm_LargeAreaEmphasis
	16	firstorder_Uniformity	61	glszm_LargeAreaHighGrayLevelEmphasis
	17	firstorder_Variance	62	glszm_LargeAreaLowGrayLevelEmphasis
	18	glcm_Autocorrelation	63	glszm_LowGrayLevelZoneEmphasis
	19	glcm_JointAverage	64	glszm_SizeZoneNonUniformity
	20	glcm_ClusterProminence	65	glszm_SizeZoneNonUniformityNormalized
	21	glcm_ClusterShade	66	glszm_SmallAreaEmphasis
	22	glcm_ClusterTendency	67	glszm_SmallAreaHighGrayLevelEmphasis
	23	glcm_Contrast	68	glszm_SmallAreaLowGrayLevelEmphasis
	24	glcm_Correlation	69	glszm_ZoneEntropy
	25	glcm_DifferenceAverage	70	glszm_ZonePercentage
	26	glcm_DifferenceEntropy	71	glszm_ZoneVariance
	27	glcm_DifferenceVariance	72	gldm_DependenceEntropy
	28	glcm_JointEnergy	73	gldm_DependenceNonUniformity
	29	glcm_JointEntropy	74	gldm_DependenceNonUniformityNormalized
	30	glcm_Imc1	75	gldm_DependenceVariance
	31	glcm_Imc2	76	gldm_GrayLevelNonUniformity
	32	glcm_Idm	77	gldm_GrayLevelVariance
	33	glcm_Idmn	78	gldm_HighGrayLevelEmphasis
	34	glcm_Id	79	gldm_LargeDependenceEmphasis
	35	glcm_Idn	80	gldm_LargeDependenceHighGrayLevelEmphasis
	36	glcm_InverseVariance	81	gldm_LargeDependenceLowGrayLevelEmphasis
	37	glcm_MaximumProbability	82	gldm_LowGrayLevelEmphasis
	38	glcm_SumEntropy	83	gldm_SmallDependenceEmphasis
	39	glcm_SumSquares	84	gldm_SmallDependenceHighGrayLevelEmphasis
	40	glrlm_GrayLevelNonUniformity	85	gldm_SmallDependenceLowGrayLevelEmphasis
	41	glrlm_GrayLevelNonUniformityNormalized	86	ngtdm_Busyness
	42	glrlm_GrayLevelVariance	87	ngtdm_Coarseness
	43	glrlm_HighGrayLevelRunEmphasis	88	ngtdm_Complexity
	44	glrlm_LongRunEmphasis	89	ngtdm_Contrast
	45	glrlm_LongRunHighGrayLevelEmphasis	90	ngtdm_Strength

[1]. Feature Groups 1: shape features; [2]. Feature Group 2: intensity histogram (first-order) features and textural (Haralick) features

Parameters of params.yaml for radiomics extraction

Parameters	Value
binWidth	25
interpolator	'sitkBSpline'
resampledPixelSpacing	[2, 2, 2]
padDistance	10
resegmentRange	[-3, 3]

resegmentMode	sigma
voxelArrayShift	1000
label	1

Supplementary Table 6. CCC of Feature Group 2 for denoised low noisy images when network trained at different epochs

Index*	Low Noisy Images	25 Epochs	50 Epochs	75 Epochs	100 Epochs
1	0.995	0.990	0.990	0.989	0.989
2	0.829	0.584	0.660	0.659	0.661
3	1.000	0.999	0.999	0.999	0.999
4	0.937	0.984	0.983	0.983	0.983
5	0.983	0.992	0.984	0.983	0.983
6	0.806	0.973	0.979	0.980	0.980
7	0.901	0.857	0.913	0.914	0.914
8	0.989	0.972	0.981	0.981	0.981
9	0.990	0.993	0.984	0.984	0.984
10	0.971	0.962	0.969	0.970	0.970
11	0.997	0.987	0.982	0.982	0.982
12	0.974	0.972	0.965	0.964	0.964
13	0.988	0.992	0.984	0.984	0.984
14	0.982	0.964	0.977	0.977	0.977
15	0.891	0.960	0.977	0.979	0.978
16	0.777	0.954	0.951	0.951	0.951
17	0.986	0.991	0.977	0.977	0.977
18	0.983	0.980	0.958	0.959	0.958
19	0.987	0.981	0.962	0.963	0.962
20	0.987	0.986	0.969	0.969	0.969
21	0.988	0.989	0.978	0.978	0.978
22	0.995	0.992	0.983	0.982	0.982
23	0.931	0.990	0.973	0.973	0.973
24	0.955	0.959	0.970	0.970	0.970
25	0.970	0.990	0.982	0.982	0.982
26	0.937	0.981	0.974	0.974	0.974
27	0.882	0.985	0.964	0.964	0.964
28	0.473	0.878	0.866	0.869	0.869
29	0.878	0.972	0.970	0.970	0.970
30	0.991	0.990	0.989	0.989	0.988
31	0.984	0.963	0.971	0.971	0.971
32	0.777	0.947	0.941	0.941	0.941
33	0.941	0.969	0.961	0.961	0.960
34	0.818	0.955	0.948	0.948	0.948
35	0.894	0.977	0.975	0.975	0.975
36	0.908	0.982	0.979	0.979	0.980
37	0.390	0.849	0.817	0.819	0.818
38	0.950	0.989	0.987	0.987	0.987
39	0.988	0.992	0.981	0.980	0.980
40	0.981	0.995	0.994	0.994	0.994
41	0.856	0.968	0.969	0.970	0.970
42	0.985	0.990	0.979	0.978	0.978
43	0.983	0.982	0.958	0.960	0.958
44	0.453	0.846	0.804	0.806	0.806
45	0.870	0.951	0.966	0.967	0.966
46	0.775	0.749	0.717	0.724	0.718
47	0.771	0.715	0.756	0.774	0.758
48	0.990	0.983	0.985	0.984	0.984
49	0.943	0.973	0.961	0.961	0.961
50	0.746	0.936	0.928	0.928	0.928
51	0.653	0.909	0.894	0.894	0.894
52	0.382	0.817	0.770	0.772	0.772
53	0.690	0.921	0.907	0.907	0.907
54	0.984	0.980	0.956	0.958	0.956

55	0.757	0.709	0.756	0.776	0.759
56	0.943	0.941	0.943	0.943	0.943
57	0.973	0.968	0.968	0.969	0.968
58	0.974	0.992	0.977	0.978	0.978
59	0.982	0.990	0.969	0.970	0.968
60	0.665	0.911	0.904	0.904	0.905
61	0.754	0.931	0.982	0.982	0.982
62	0.621	0.839	0.797	0.797	0.798
63	0.719	0.784	0.792	0.800	0.787
64	0.971	0.981	0.972	0.972	0.971
65	0.813	0.801	0.852	0.842	0.865
66	0.809	0.814	0.855	0.850	0.865
67	0.976	0.988	0.965	0.966	0.964
68	0.688	0.783	0.798	0.810	0.787
69	0.961	0.952	0.977	0.976	0.977
70	0.984	0.987	0.990	0.990	0.990
71	0.665	0.911	0.905	0.905	0.905
72	0.982	0.943	0.972	0.971	0.972
73	0.901	0.951	0.939	0.939	0.939
74	0.951	0.965	0.982	0.980	0.982
75	0.354	0.766	0.762	0.764	0.762
76	0.902	0.978	0.979	0.979	0.979
77	0.987	0.994	0.984	0.984	0.985
78	0.981	0.987	0.966	0.967	0.965
79	0.436	0.840	0.810	0.812	0.811
80	0.571	0.848	0.942	0.943	0.942
81	0.634	0.768	0.709	0.711	0.710
82	0.787	0.846	0.816	0.838	0.825
83	0.976	0.981	0.986	0.985	0.986
84	0.978	0.990	0.976	0.977	0.975
85	0.802	0.926	0.881	0.891	0.880
86	0.994	0.987	0.979	0.977	0.979
87	0.981	0.993	0.994	0.994	0.991
88	0.922	0.974	0.949	0.950	0.948
89	0.945	0.972	0.966	0.964	0.965
90	0.987	0.985	0.985	0.985	0.983

* Index in Supplementary Table 5 Feature Group 2

Supplementary Table 7. CCC of Feature Group 2 for denoised low noisy images when network trained at different epochs

Index*	High Noisy Images	25 Epochs	50 Epochs	75 Epochs	100 Epochs
1	0.983	0.991	0.989	0.989	0.989
2	0.630	0.600	0.768	0.765	0.763
3	1.000	0.999	0.999	0.999	0.999
4	0.642	0.976	0.988	0.988	0.989
5	0.931	0.993	0.984	0.984	0.984
6	0.385	0.931	0.929	0.935	0.935
7	0.846	0.888	0.911	0.912	0.912
8	0.987	0.974	0.982	0.981	0.981
9	0.954	0.994	0.985	0.985	0.985
10	0.955	0.970	0.967	0.967	0.967
11	0.984	0.989	0.982	0.983	0.983
12	0.936	0.978	0.968	0.969	0.969
13	0.945	0.993	0.984	0.984	0.984
14	0.982	0.967	0.978	0.977	0.977
15	0.490	0.912	0.926	0.931	0.932
16	0.336	0.917	0.952	0.953	0.953
17	0.981	0.991	0.978	0.977	0.977
18	0.985	0.982	0.959	0.961	0.961
19	0.974	0.984	0.965	0.966	0.966
20	0.985	0.983	0.963	0.962	0.962
21	0.983	0.987	0.974	0.974	0.974
22	0.991	0.993	0.982	0.981	0.981
23	0.908	0.988	0.975	0.975	0.975
24	0.849	0.967	0.978	0.978	0.978
25	0.781	0.991	0.990	0.990	0.990
26	0.567	0.980	0.987	0.987	0.987
27	0.869	0.978	0.958	0.957	0.957
28	0.095	0.761	0.851	0.854	0.854
29	0.426	0.953	0.976	0.976	0.976
30	0.972	0.995	0.994	0.994	0.994
31	0.938	0.987	0.989	0.989	0.989
32	0.278	0.935	0.961	0.962	0.962
33	0.755	0.974	0.964	0.964	0.965
34	0.338	0.946	0.969	0.969	0.969
35	0.552	0.975	0.972	0.971	0.972
36	0.370	0.978	0.987	0.987	0.987
37	0.065	0.755	0.845	0.846	0.848
38	0.699	0.981	0.991	0.991	0.991
39	0.982	0.992	0.980	0.980	0.980
40	0.826	0.995	0.997	0.997	0.997
41	0.414	0.948	0.969	0.970	0.970
42	0.981	0.990	0.976	0.976	0.976
43	0.982	0.983	0.962	0.964	0.963
44	0.136	0.782	0.852	0.854	0.855
45	0.777	0.917	0.909	0.909	0.909
46	0.413	0.748	0.857	0.855	0.853
47	0.668	0.671	0.786	0.779	0.780
48	0.881	0.990	0.990	0.990	0.991
49	0.843	0.980	0.989	0.989	0.989
50	0.283	0.927	0.955	0.956	0.957
51	0.224	0.894	0.932	0.934	0.934
52	0.109	0.729	0.816	0.818	0.819
53	0.242	0.910	0.944	0.945	0.945
54	0.967	0.982	0.966	0.968	0.968

55	0.697	0.663	0.775	0.767	0.768
56	0.781	0.974	0.980	0.980	0.980
57	0.684	0.971	0.977	0.976	0.976
58	0.968	0.992	0.980	0.980	0.980
59	0.936	0.985	0.979	0.981	0.981
60	0.137	0.843	0.884	0.886	0.889
61	0.343	0.845	0.908	0.899	0.900
62	0.057	0.824	0.890	0.894	0.900
63	0.327	0.766	0.835	0.850	0.846
64	0.657	0.990	0.992	0.992	0.992
65	0.717	0.804	0.813	0.814	0.819
66	0.722	0.803	0.822	0.826	0.832
67	0.940	0.985	0.975	0.977	0.976
68	0.329	0.728	0.811	0.822	0.817
69	0.711	0.947	0.974	0.972	0.973
70	0.766	0.988	0.992	0.991	0.991
71	0.137	0.843	0.884	0.886	0.889
72	0.943	0.964	0.976	0.978	0.978
73	0.621	0.972	0.987	0.987	0.987
74	0.662	0.972	0.980	0.982	0.980
75	0.084	0.722	0.812	0.815	0.818
76	0.636	0.962	0.978	0.978	0.978
77	0.980	0.994	0.983	0.983	0.983
78	0.983	0.985	0.968	0.971	0.970
79	0.113	0.798	0.869	0.871	0.872
80	0.230	0.768	0.834	0.837	0.838
81	0.156	0.853	0.904	0.903	0.904
82	0.721	0.760	0.799	0.800	0.799
83	0.738	0.983	0.988	0.988	0.988
84	0.870	0.984	0.981	0.982	0.982
85	0.799	0.836	0.849	0.843	0.841
86	0.939	0.988	0.974	0.975	0.975
87	0.983	0.990	0.992	0.992	0.991
88	0.904	0.976	0.963	0.963	0.963
89	0.926	0.949	0.969	0.969	0.970
90	0.988	0.990	0.989	0.990	0.991

* Index in Supplementary Table 5 Feature Group 2

Supplementary Table 8. RMSE, content loss and ratio of poor, medium, and good reproducibility radiomic features for images denoised by the Cycle GAN trained for different numbers of epochs

Training length / Noisy images	25 Epochs	50 Epochs	75 Epochs	100 Epochs
Low-noise Images				
RMSE	0.0178	0.0169	0.0172	0.0170
Content loss	0.0229	0.216	0.0217	0.0216
CCCs ≥ 0.85	81%	84%	82%	84%
0.65≤CCCs<0.85	18%	16%	18%	16%
CCCs<0.65	1%	0%	0%	0%
High-noise Images				
RMSE	0.0193	0.0177	0.0175	0.0181
Content loss	0.256	0.0241	0.0248	0.0245
CCCs > 0.85	77%	86%	86%	86%
0.65≤CCCs<0.85	22%	14%	14%	14%
CCCs<0.65	1%	0%	0%	0%

Supplementary Table 9. RMSE, content loss and ratio of poor, medium, and good reproducibility radiomic features for images denoised by the Cycle GAN trained without training strategy for different numbers of epochs

Noisy images \ Training length	25 Epochs	50 Epochs	75 Epochs	100 Epochs
Low-noise Images				
RMSE	0.0199	0.0167	0.167	0.0167
Content loss	0.0830	0.0258	0.0258	0.0258
CCCs ≥ 0.85	61%	86%	86%	86%
0.65≤CCCs<0.85	36%	13%	13%	13%
CCCs<0.65	4%	1%	1%	1%
High-noise Images				
RMSE	0.0201	0.0188	0.0188	0.0188
Content loss	0.0877	0.0263	0.0263	0.0263
CCCs > 0.85	74%	86%	87%	84%
0.65≤CCCs<0.85	25%	11%	11%	12%
CCCs<0.65	1%	3%	2%	3%

Supplementary Table 10. Importance of features in 4-year pre-treatment survival predication models (Original Radiomics features)

Rank	Features	Rank	Features
1	glszm_LargeAreaLowGrayLevelEmphasis	53	firstorder_InterquartileRange
2	ngtdm_Coarseness	54	glcm_Correlation
3	gldm_GrayLevelVariance	55	glcm_Idn
4	firstorder_Entropy	56	glszm_ZonePercentage
5	shape_MinorAxisLength	57	gldm_SmallDependenceEmphasis
6	glrlm_GrayLevelNonUniformityNormalized	58	shape_Maximum3DDiameter
7	glszm_LargeAreaHighGrayLevelEmphasis	59	firstorder_Skewness
8	glcm_JointEntropy	60	glszm_SmallAreaEmphasis
9	glrlm_RunLengthNonUniformityNormalized	61	glszm_SizeZoneNonUniformityNormalized
10	glszm_LowGrayLevelZoneEmphasis	62	firstorder_RobustMeanAbsoluteDeviation
11	glszm_GrayLevelNonUniformityNormalized	63	glcm_Autocorrelation
12	shape_SurfaceVolumeRatio	64	glcm_Idmn
13	glcm_SumEntropy	65	glcm_ClusterShade
14	gldm_LargeDependenceLowGrayLevelEmphasis	66	glszm_GrayLevelVariance
15	glszm_SizeZoneNonUniformityNormalized	67	glrlm_LongRunLowGrayLevelEmphasis
16	glrlm_ShortRunEmphasis	68	shape_Maximum2DDiameterColumn
17	gldm_DependenceNonUniformityNormalized	69	glcm_InverseVariance
18	gldm_GrayLevelNonUniformity	70	glcm_JointAverage
19	gldm_LargeDependenceHighGrayLevelEmphasis	71	firstorder_RootMeanSquared
20	shape_MajorAxisLength	72	gldm_SmallDependenceHighGrayLevelEmphasis
21	gldm_LowGrayLevelEmphasis	73	ngtdm_Contrast
22	glcm_Idm	74	firstorder_Minimum
23	glrlm_LongRunHighGrayLevelEmphasis	75	glszm_SmallAreaLowGrayLevelEmphasis
24	firstorder_Maximum	76	glrlm_LowGrayLevelRunEmphasis
25	shape_Maximum2DDiameterColumn	77	glrlm_ShortRunHighGrayLevelEmphasis
26	glcm_Imc2	78	firstorder_MeanAbsoluteDeviation
27	shape_Maximum2DDiameterSlice	79	glszm_SmallAreaHighGrayLevelEmphasis
28	shape_Sphericity	80	ngtdm_Complexity
29	gldm_DependenceEntropy	81	glrlm_GrayLevelVariance
30	shape_Elongation	82	shape_SurfaceVolumeRatio
31	glrlm_RunVariance	83	glrlm_RunEntropy
32	glcm_Imc1	84	glszm_HighGrayLevelZoneEmphasis
33	glrlm_LongRunLowGrayLevelEmphasis	85	gldm_DependenceEntropy
34	firstorder_Variance	86	gldm_SmallDependenceLowGrayLevelEmphasis
35	glrlm_ShortRunHighGrayLevelEmphasis	87	glrlm_ShortRunLowGrayLevelEmphasis
36	glrlm_RunPercentage	88	gldm_HighGrayLevelEmphasis
37	glrlm_GrayLevelVariance	89	gldm_DependenceNonUniformityNormalized
38	firstorder_Mean	90	glcm_DifferenceVariance
39	firstorder_Minimum	91	firstorder_Variance
40	glcm_SumSquares	92	firstorder_10Percentile
41	glszm_ZonePercentage	93	shape_Flatness
42	shape_Flatness	94	gldm_LowGrayLevelEmphasis
43	shape_Maximum3DDiameter	95	glcm_ClusterTendency
44	glcm_ClusterShade	96	firstorder_90Percentile
45	firstorder_InterquartileRange	97	glszm_LowGrayLevelZoneEmphasis
46	glcm_DifferenceVariance	98	glcm_DifferenceAverage
47	firstorder_RobustMeanAbsoluteDeviation	99	gldm_GrayLevelVariance
48	glcm_Idmn	100	glszm_GrayLevelNonUniformityNormalized
49	glcm_Idn	101	glrlm_HighGrayLevelRunEmphasis
50	ngtdm_Complexity	102	glcm_Contrast
51	Age	103	glcm_SumSquares
52	shape_SurfaceArea	104	firstorder_InterquartileRange

Supplementary Table 11. Importance of features in 4-year pre-treatment survival predication models (EDNs De-noised Radiomics features)

Rank	Features	Rank	Features
1	glszm_LargeAreaLowGrayLevelEmphasis	53	firstorder_Uniformity
2	gldm_GrayLevelVariance	54	shape_SurfaceArea
3	glrlm_GrayLevelNonUniformityNormalized	55	glszm_HighGrayLevelZoneEmphasis
4	gldm_LargeDependenceHighGrayLevelEmphasis	56	glrlm_ShortRunLowGrayLevelEmphasis
5	gldm_GrayLevelNonUniformity	57	glszm_GrayLevelVariance
6	firstorder_Entropy	58	glszm_LargeAreaEmphasis
7	glcm_JointEntropy	59	glcm_Imc2
8	ngtdm_Coarseness	60	firstorder_Mean
9	glcm_SumEntropy	61	glcm_ClusterTendency
10	glrlm_ShortRunEmphasis	62	glcm_Contrast
11	shape_MinorAxisLength	63	glcm_DifferenceEntropy
12	gldm_DependenceEntropy	64	firstorder_Median
13	glszm_LargeAreaHighGrayLevelEmphasis	65	firstorder_RobustMeanAbsoluteDeviation
14	glrlm_LongRunHighGrayLevelEmphasis	66	glszm_SmallAreaLowGrayLevelEmphasis
15	shape_SurfaceVolumeRatio	67	firstorder_10Percentile
16	glszm_GrayLevelNonUniformityNormalized	68	glcm_MaximumProbability
17	gldm_LargeDependenceLowGrayLevelEmphasis	69	shape_Maximum2DDiameterRow
18	gldm_DependenceVariance	70	glcm_JointEnergy
19	glcm_Idm	71	glrlm_LongRunEmphasis
20	glrlm_RunLengthNonUniformityNormalized	72	shape_LeastAxisLength
21	glszm_LowGrayLevelZoneEmphasis	73	gldm_SmallDependenceEmphasis
22	glrlm_RunVariance	74	shape_Maximum3DDiameter
23	firstorder_Variance	75	gldm_LargeDependenceEmphasis
24	shape_MajorAxisLength	76	glszm_ZonePercentage
25	shape_Elongation	77	firstorder_90Percentile
26	gldm_LowGrayLevelEmphasis	78	glcm_InverseVariance
27	glszm_SmallAreaEmphasis	79	gldm_SmallDependenceHighGrayLevelEmphasis
28	glrlm_ShortRunHighGrayLevelEmphasis	80	firstorder_Energy
29	glszm_SizeZoneNonUniformityNormalized	81	glrlm_RunEntropy
30	shape_Maximum2DDiameterSlice	82	glcm_DifferenceAverage
31	glszm_ZoneEntropy	83	firstorder_MeanAbsoluteDeviation
32	firstorder_Maximum	84	glszm_GrayLevelNonUniformity
33	gldm_DependenceNonUniformityNormalized	85	ngtdm_Busyness
34	glrlm_GrayLevelVariance	86	glrlm_LowGrayLevelRunEmphasis
35	glrlm_RunPercentage	87	glcm_Id
36	shape_Maximum2DDiameterColumn	88	firstorder_Skewness
37	glcm_Imc1	89	glrlm_GrayLevelNonUniformity
38	shape_Sphericity	90	glcm_ClusterProminence
39	ngtdm_Contrast	91	glcm_Autocorrelation
40	glszm_SmallAreaHighGrayLevelEmphasis	92	gldm_HighGrayLevelEmphasis
41	firstorder_InterquartileRange	93	Age
42	glcm_Idmn	94	glszm_ZoneVariance
43	glszm_SizeZoneNonUniformity	95	glcm_JointAverage
44	ngtdm_Complexity	96	glcm_ClusterShade
45	firstorder_Kurtosis	97	glrlm_RunLengthNonUniformity
46	glcm_SumSquares	98	ngtdm_Strength
47	glrlm_LongRunLowGrayLevelEmphasis	99	glrlm_HighGrayLevelRunEmphasis
48	glcm_Idn	100	gldm_DependenceNonUniformity
49	glcm_DifferenceVariance	101	gldm_SmallDependenceLowGrayLevelEmphasis
50	firstorder_RootMeanSquared	102	shape_MeshVolume
51	glcm_Correlation	103	firstorder_Range
52	shape_Flatness	104	firstorder_Uniformity

Supplementary Table 12. Importance of features in 4-year pre-treatment survival predication models (CGAN De-noised Radiomics features)

Rank	Features	Rank	Features
1	glszm_LargeAreaLowGrayLevelEmphasis	53	glcm_DifferenceAverage
2	glrlm_GrayLevelNonUniformityNormalized	54	firstorder_Uniformity
3	gldm_GrayLevelVariance	55	glcm_DifferenceVariance
4	firstorder_Entropy	56	glszm_SmallAreaLowGrayLevelEmphasis
5	gldm_GrayLevelNonUniformity	57	glrlm_LongRunLowGrayLevelEmphasis
6	ngtdm_Coarseness	58	glcm_MaximumProbability
7	glcm_JointEntropy	59	ngtdm_Strength
8	shape_MinorAxisLength	60	glcm_JointAverage
9	gldm_DependenceEntropy	61	glcm_Correlation
10	glszm_LargeAreaHighGrayLevelEmphasis	62	gldm_HighGrayLevelEmphasis
11	gldm_LargeDependenceHighGrayLevelEmphasis	63	firstorder_Range
12	glszm_LowGrayLevelZoneEmphasis	64	glszm_ZonePercentage
13	glrlm_RunLengthNonUniformityNormalized	65	shape_Maximum3DDiameter
14	glszm_GrayLevelNonUniformityNormalized	66	shape_Maximum2DDiameterRow
15	glrlm_ShortRunEmphasis	67	glcm_Autocorrelation
16	glcm_SumEntropy	68	glcm_ClusterProminence
17	shape_SurfaceVolumeRatio	69	glszm_SizeZoneNonUniformity
18	glrlm_LongRunHighGrayLevelEmphasis	70	gldm_SmallDependenceHighGrayLevelEmphasis
19	gldm_LargeDependenceLowGrayLevelEmphasis	71	firstorder_RobustMeanAbsoluteDeviation
20	gldm_DependenceVariance	72	glcm_DifferenceEntropy
21	glcm_Idm	73	firstorder_RootMeanSquared
22	firstorder_Variance	74	glszm_GrayLevelVariance
23	glrlm_RunVariance	75	firstorder_90Percentile
24	gldm_LowGrayLevelEmphasis	76	glrlm_GrayLevelNonUniformity
25	glszm_SizeZoneNonUniformityNormalized	77	gldm_SmallDependenceLowGrayLevelEmphasis
26	shape_MajorAxisLength	78	glszm_ZoneEntropy
27	glrlm_ShortRunHighGrayLevelEmphasis	79	glrlm_RunLengthNonUniformity
28	shape_Elongation	80	glcm_ClusterTendency
29	gldm_DependenceNonUniformityNormalized	81	firstorder_Median
30	glcm_Imc1	82	gldm_DependenceNonUniformity
31	shape_Maximum2DDiameterSlice	83	glcm_InverseVariance
32	shape_Maximum2DDiameterColumn	84	firstorder_Skewness
33	glrlm_RunPercentage	85	glszm_ZoneVariance
34	glszm_HighGrayLevelZoneEmphasis	86	firstorder_10Percentile
35	glrlm_GrayLevelVariance	87	shape_MeshVolume
36	glrlm_ShortRunLowGrayLevelEmphasis	88	firstorder_Kurtosis
37	firstorder_Maximum	89	ngtdm_Contrast
38	shape_Flatness	90	glcm_JointEnergy
39	shape_Sphericity	91	glszm_GrayLevelNonUniformity
40	ngtdm_Complexity	92	firstorder_Mean
41	glcm_Imc2	93	firstorder_MeanAbsoluteDeviation
42	glszm_SmallAreaEmphasis	94	firstorder_Minimum
43	glcm_Idmn	95	shape_LeastAxisLength
44	glrlm_LongRunEmphasis	96	Age
45	glcm_ClusterShade	97	glcm_Contrast
46	glszm_SmallAreaHighGrayLevelEmphasis	98	gldm_SmallDependenceEmphasis
47	glszm_LargeAreaEmphasis	99	glrlm_RunEntropy
48	glcm_Idn	100	ngtdm_Busyness
49	gldm_LargeDependenceEmphasis	101	glrlm_LowGrayLevelRunEmphasis
50	firstorder_InterquartileRange	102	firstorder_Energy
51	glrlm_HighGrayLevelRunEmphasis	103	glcm_Id
52	glcm_SumSquares	104	glcm_DifferenceAverage

Supplementary Table 13. Importance of features in 4-year pre-treatment survival predication models (Cycle-GAN De-noised Radiomics features)

Rank	Features	Rank	Features
1	glrlm_GrayLevelNonUniformityNormalized	53	shape_Flatness
2	glszm_LargeAreaLowGrayLevelEmphasis	54	shape_LeastAxisLength
3	gldm_GrayLevelVariance	55	shape_SurfaceArea
4	firstorder_Entropy	56	firstorder_Energy
5	shape_MinorAxisLength	57	glcm_Correlation
6	ngtdm_Coarseness	58	firstorder_10Percentile
7	glcm_JointEntropy	59	firstorder_MeanAbsoluteDeviation
8	glrlm_RunLengthNonUniformityNormalized	60	Age
9	gldm_DependenceEntropy	61	shape_Maximum2DDiameterRow
10	glszm_LargeAreaHighGrayLevelEmphasis	62	glszm_HighGrayLevelZoneEmphasis
11	glszm_LowGrayLevelZoneEmphasis	63	firstorder_RobustMeanAbsoluteDeviation
12	glszm_GrayLevelNonUniformityNormalized	64	firstorder_Range
13	glszm_SizeZoneNonUniformityNormalized	65	glszm_LargeAreaEmphasis
14	gldm_LargeDependenceHighGrayLevelEmphasis	66	gldm_DependenceNonUniformity
15	shape_SurfaceVolumeRatio	67	glcm_ClusterTendency
16	glszm_ZoneEntropy	68	glcm_JointEnergy
17	glrlm_ShortRunEmphasis	69	firstorder_Skewness
18	gldm_GrayLevelNonUniformity	70	firstorder_RootMeanSquared
19	shape_Elongation	71	firstorder_Median
20	gldm_DependenceNonUniformityNormalized	72	glrlm_ShortRunLowGrayLevelEmphasis
21	glrlm_LongRunHighGrayLevelEmphasis	73	firstorder_Kurtosis
22	glcm_SumEntropy	74	glcm_MaximumProbability
23	shape_MajorAxisLength	75	glcm_ClusterShade
24	glcm_Idm	76	glcm_ClusterProminence
25	firstorder_Variance	77	glrlm_RunLengthNonUniformity
26	glszm_SizeZoneNonUniformity	78	glszm_ZonePercentage
27	gldm_LargeDependenceLowGrayLevelEmphasis	79	firstorder_90Percentile
28	firstorder_Maximum	80	glszm_SmallAreaLowGrayLevelEmphasis
29	shape_Maximum2DDiameterSlice	81	glszm_ZoneVariance
30	glrlm_RunVariance	82	gldm_SmallDependenceLowGrayLevelEmphasis
31	glrlm_ShortRunHighGrayLevelEmphasis	83	glcm_InverseVariance
32	gldm_DependenceVariance	84	gldm_SmallDependenceEmphasis
33	shape_Maximum2DDiameterColumn	85	glszm_GrayLevelNonUniformity
34	glcm_Imc1	86	glcm_JointAverage
35	shape_Sphericity	87	glrlm_RunEntropy
36	gldm_LowGrayLevelEmphasis	88	glrlm_LongRunEmphasis
37	glrlm_RunPercentage	89	shape_Maximum3DDiameter
38	glrlm_GrayLevelVariance	90	glcm_DifferenceAverage
39	glcm_Id	91	ngtdm_Busyness
40	glcm_Idmn	92	glrlm_HighGrayLevelRunEmphasis
41	glszm_SmallAreaEmphasis	93	gldm_LargeDependenceEmphasis
42	glrlm_LongRunLowGrayLevelEmphasis	94	glcm_Contrast
43	firstorder_Uniformity	95	gldm_HighGrayLevelEmphasis
44	firstorder_Minimum	96	glcm_Autocorrelation
45	glszm_SmallAreaHighGrayLevelEmphasis	97	glszm_GrayLevelVariance
46	glrlm_LowGrayLevelRunEmphasis	98	shape_MeshVolume
47	firstorder_InterquartileRange	99	glcm_DifferenceEntropy
48	ngtdm_Complexity	100	glcm_Imc2
49	glcm_SumSquares	101	ngtdm_Contrast
50	glcm_Idn	102	glrlm_GrayLevelNonUniformity
51	ngtdm_Strength	103	gldm_SmallDependenceHighGrayLevelEmphasis
52	firstorder_Mean	104	shape_Flatness

Supplementary Method 1: Noise

The high quality NSCLC-Radiomics collection [1] (hereafter called LUNG 1), which contains CT scans of 422 non-small cell lung cancer (NSCLC) patients, as our experimental dataset. These CT scans included annotations drawn by specialist radiation oncologists that delineate a region of interest (ROI), the gross tumor volume. ROIs were necessary to be able to compute radiomic features. The CT images for which the dose level ('parameter exposure' in DICOM metadata) was missing (n=200) were excluded from further analyses. We considered CT images scanned at 400 milliampere-seconds (mAs) and above as full dose CT (n=157, the index of LUNG 1 patients included in the experiments can be found in Supplementary Table 1, supplementary materials are available: https://gitlab.com/UM-CDS/low-dose-ct-denoising/-/branches). These data were be used for training (n=40, 4260 frames) and testing (n=117, 13423 frames). Conversely, we designated CT images scanned at 50 mAs as low dose CT, taking the same definition as a prior Low Dose CT Grand Challenge [2][3].

As mentioned, training of EDNs and CGANs require paired images, in our case, pairs of matching low dose and full dose CT scans. However, LUNG 1 contains no paired images, thus we simulated the noisy degradation present in low dose CT images by introducing noise using the method proposed in literature [2][3]. In these, the authors had mimicked CT scanners' behavior by adding noise with a normal distribution into a sinogram (by Radon transform) and reconstructed the CT image from the modified sinogram to obtain simulated noisy images. We used a similar method to add noise in the original sinogram as follows:

$$z_i = (1 + b_i)e^i + r_i, i = 1, \ldots, I, b_i \sim N(\mu, \sigma) \quad (1)$$

where z_i is the measurement along the i-th ray path; r_i is the read-out error; e^i represents the original line integral of attenuation coefficients along the i-th ray path; and, b_i is the black scanner factor, which follows a normal distribution. The intensity of noise added to the image can be controlled through the parameter b_i.

To simulate low dose CT images (scanned with 50mAs) from full dose CT images (scanned with 400 mAs), we first measured the noise intensity introduced in images with lower doses by scanning a Gammex 467 CT phantom (Middleton, WI, USA) using a Philips Brilliance Big Bore CT at two dose levels (400 mAs and 50 mAs)[4]. The signal-to-noise ratio (SNR) of the real phantom dataset was 19.7 dB (95%CI [17.8, 21.6]). We thus estimated that a σ value of 0.0035 best estimated the noise in 50 mAs CT images when generated from 400 mAs images. The SNR in the simulated low-noise images was 18.3 (95%CI, [16.9, 20.1]) dB, close to the real value. To assess the reproducibility of radiomic features with noise of different intensities, we added stronger noise (25 times noise power) by setting σ to 0.0068 to mimic CT images with stronger noise (referred to as simulated high-noise images hereafter). The SNR in the simulated high-noise images had thus reduced to 6.0 (95%CI, [5.9,6.1]) dB. Additionally, extraneous noise introduced by the Radon transform and inverse Radon transform was filtered from the simulated images. A comparison of noise in simulated images and in real phantom scans is shown in following Figure 1, the intensity of noise in real phantom is 17.1 dB and average noise power spectra density within whole image is 45.8 W/Hz. The intensity of noise in simulated low-noise images is 19.4 dB and average noise power spectra density within whole image is 3.6 W/Hz, intensity of noise in simulated high-noise images is 6.1 dB and average noise power spectra density within whole image is 6.0 W/Hz.

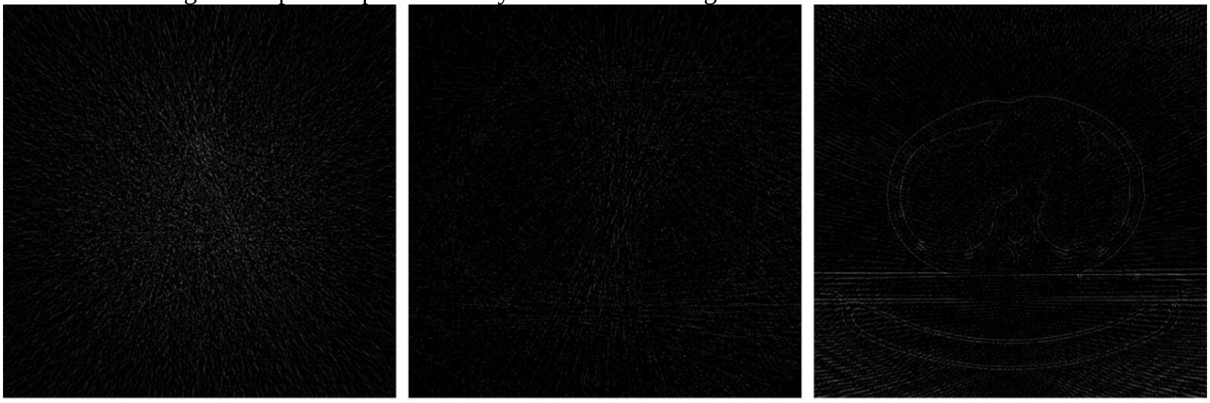

(a) SNR=17.1 dB Average PSD=45.8 W/Hz
(b) SNR=19.4 dB Average PSD=3.6 W/Hz
(c) SNR=6.1 dB Average PSD=6.0 W/Hz

Figure 1.A comparison of noise in simulated images and in a real phantom image. (a) real phantom image; (b) simulated low-noise image; (c) simulated high-noise image.

Reference

[1] Aerts, H. J. W. L., Wee, L., Rios Velazquez, E., Leijenaar, R. T. H., Parmar, C., Grossmann, P., … Lambin, P. (2019). Data From NSCLC-Radiomics [Data set]. The Cancer Imaging Archive. https://doi.org/10.7937/K9/TCIA.2015.PF0M9REI

[2] Chen, Hu, et al. "Low-dose CT with a residual encoder-decoder convolutional neural network." IEEE transactions on medical imaging 36.12 (2017): 2524-2535.

[3] McCollough, Cynthia H., et al. "Low-dose CT for the detection and classification of metastatic liver lesions: Results of the 2016 Low Dose CT Grand Challenge." Medical physics 44.10 (2017): e339-e352.

[4] Zhovannik, Ivan, et al. "Learning from scanners: Bias reduction and feature correction in radiomics." Clinical and translational radiation oncology 19 (2019): 33-38.
